# New hydrazonoindolin-2-ones: Synthesis, exploration of the possible anti-proliferative mechanism of action and encapsulation into PLGA microspheres

**DOI:** 10.1371/journal.pone.0181241

**Published:** 2017-07-25

**Authors:** Mohamed I. Attia, Wagdy M. Eldehna, Samar A. Afifi, Adam B. Keeton, Gary A. Piazza, Hatem A. Abdel-Aziz

**Affiliations:** 1 Department of Pharmaceutical Chemistry, College of Pharmacy, King Saud University, Riyadh, Saudi Arabia; 2 Medicinal and Pharmaceutical Chemistry Department, Pharmaceutical and Drug Industries Research Division, National Research Centre (ID: 60014618), Dokki, Giza, Egypt; 3 Department of Pharmaceutical Chemistry, Faculty of Pharmacy, Kafrelsheikh University, Kafrelsheikh, Egypt; 4 Department of Pharmaceutics, National Organization for Drug Control and Research, Giza, Egypt; 5 Department of Pharmaceutics, College of Pharmacy, King Saud University, Riyadh, Saudi Arabia; 6 Department of Oncologic Sciences and Pharmacology, Drug Discovery Research Center, Mitchell Cancer Institute, University of South Alabama, Mobile, AL, United States of America; 7 Department of Applied Organic Chemistry, National Res earch Centre, (ID: 60014618), Dokki, Giza, Egypt; Texas Technical University Health Sciences Center, UNITED STATES

## Abstract

The synthesis and molecular characterization of new isatin-based hydrazonoindolin-2-ones **4a-o** and **7a-e** are reported. The *in vitro* anti-proliferative potential of the synthesized compounds **4a-o** and **7a-e** was examined against HT-29 (colon), ZR-75 (breast) and A549 (lung) human cancer cell lines. Compounds **7b**, **7d** and **7e** were the most active congeners against the tested human cancer cell lines with average IC_50_ values of 4.77, 3.39 and 2.37 μM, respectively, as compared with the reference isatin-based drug, sunitinib, which exhibited an average IC_50_ value of 8.11 μM. Compound **7e** was selected for further pharmacological evaluation in order to gain insight into its possible mechanism of action. It increased caspase 3/7 activity by 2.4- and 1.85-fold between 4 and 8 h of treatment, respectively, at 10 μM and it caused a decrease in the percentage of cells in the G1 phase of the cell cycle with a corresponding increase in the S-phase. In addition, compound **7e** increased phosphorylated tyrosine (p-Tyr) levels nearly two-fold with an apparent IC_50_ value of 3.8 μM. The **7e**-loaded PLGA microspheres were prepared using a modified emulsion-solvent diffusion method. The average encapsulation efficiency of the **7e**-loaded PLGA microspheres was 85% ± 1.3. While, the *in vitro* release profile of the **7e**-loaded microspheres was characterized by slow and continuous release of compound **7e** during 21 days and the release curve was fitted to zero order kinetics. Incorporation of **7e** into PLGA microspheres improved its *in vitro* anti-proliferative activity toward the human cancer cell line A549 after 120 h incubation period with an IC_50_ value less than 0.8 μM.

## Introduction

Cancer is one of the most formidable health burdens with increasing annual frequency worldwide [1]. In spite of precluding many of its causative risk factors, cancer still gives rise to about 550,000 deaths in the world every year and is the second leading cause of death next to heart disease [2]. The currently available cancer therapies usually suffer from poor selectivity between normal and invaded cells, with serious side effects and the development of drug resistance [3]. Therefore, the continued search to identify novel antitumor compounds bestowed with low toxicity and minimum side effects remains critically important.

**Fig 1:**
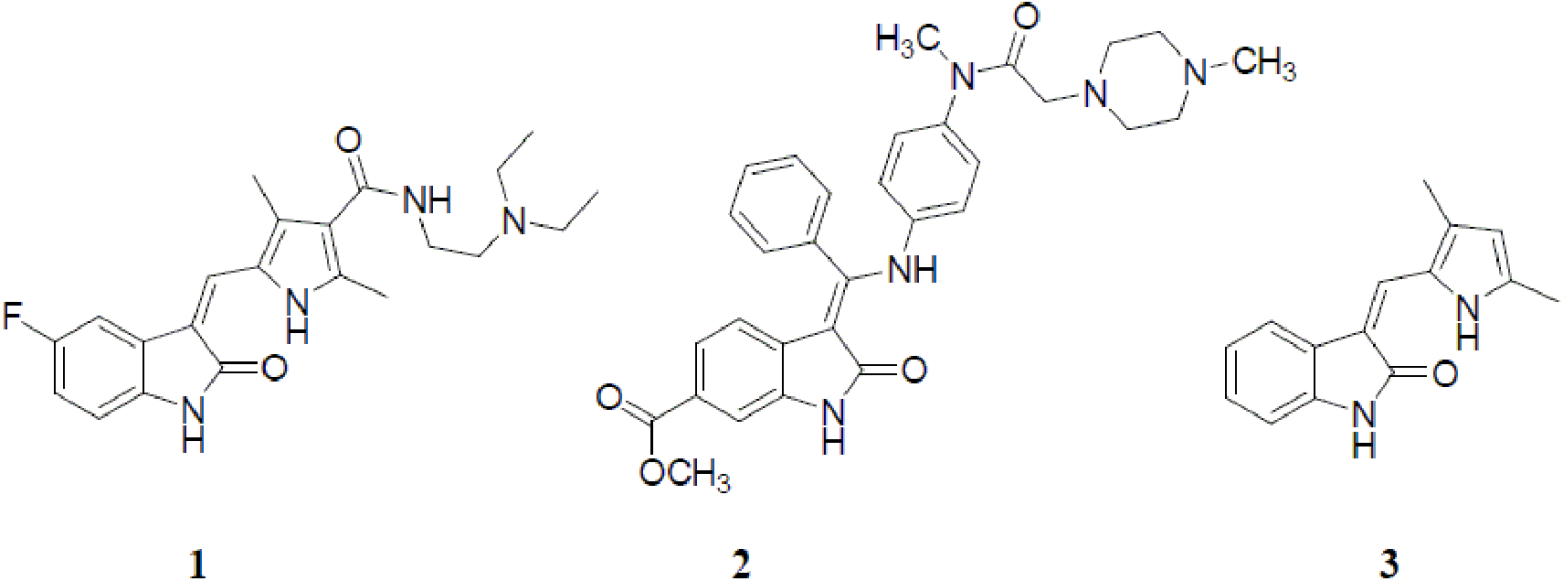
Chemical structures of some clinically used isatine-based anticancer agents.

The 1*H*-Indole-2,3-dione (isatin) moiety constitutes the backbone of a number of agrochemicals, dyes and bioactive molecules, owing to its appropriate size and unique electronic properties [4–7]. Various 3-substituted isatins have been utilized as anticancer drugs or drug surrogates. Example of this class of isatin derivatives is sunitinib (**1**, SU11248, Sutent^TM^, Pfizer, Inc., Fig 1) which was clinically approved in 2006 and is used now as the standard first-line treatment for the management of gastrointestinal stromal cancers and renal cell carcinoma due to its multi-targeting tyrosine kinase inhibiting activity [8]. Nintedanib (**2**, Fig 1) is another potential anticancer agent of this class that possesses triple angiokinase inhibiting activity toward vascular endothelial growth factor receptors (VEGFRs),platelet-derived growth factor receptors(PDGFRs) and fibroblast growth factor receptors (FGFRs)[9]. Semaxanib (**3**, Fig 1) was developed as a new anticancer candidate with multiple tyrosinekinase receptor inhibitor activity with GI_50_ values of less than 10 nM against 46 out of 53 NCI cell lines, but it has been discontinued in clinical trials owing to its dangerous side effects [10, 11].

Schiff bases of isatin are acknowledged to have a broad spectrum of biological activities including anticonvulsant [12], antibacterial [13], antifungal [14], anti-HIV [15], antiviral [16] and anticancer activity [17–19].

**Fig 2:**
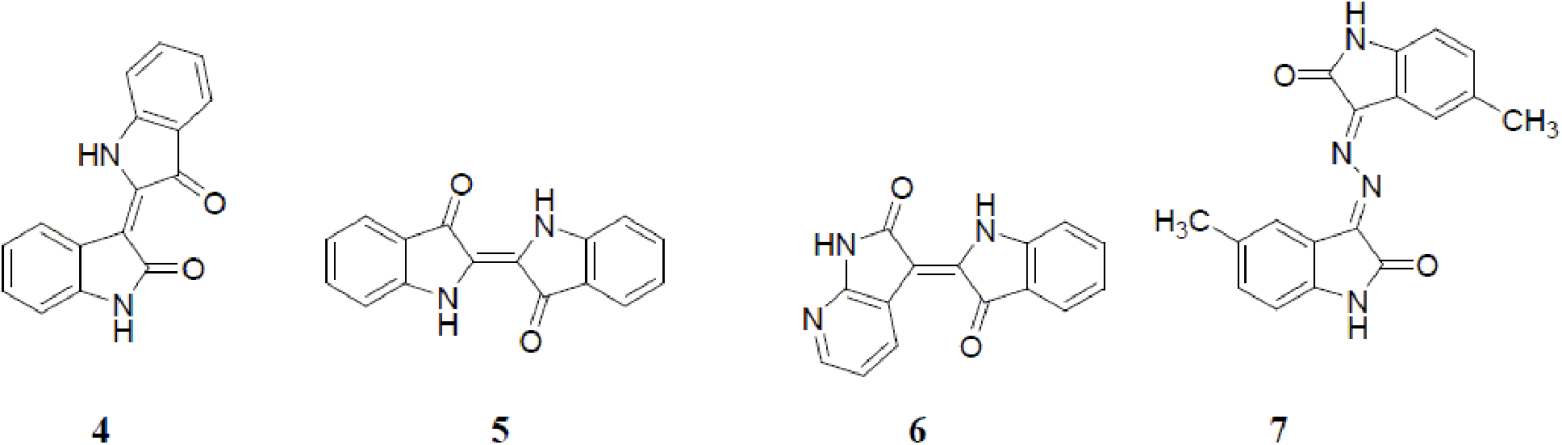
Chemical structures of some bioactive bis-isatin compounds.

Indirubin (**4**, Fig 2) is a bis-isatin isomer of the widely used colorant indigo (**5**, Fig 2), and it is the active ingredient of a traditional Chinese remedy (Danggui Longhui Wan) that is used for the treatment of chronic myelogenous leukemia (CML) [20]. Moreover, indirubin (**4**) inhibits both glycogen synthase kinase 3 beta (GSK-3β) and cyclin-dependent kinases (CDKs) *via* binding to the ATP pocket with IC_50_ values of 0.190 and 0.075 mM, respectively [21, 22]. Azaindirubin (**6**, Fig 2) was developed to overcome the poor water solubility and hence low bioavailability of indirubin (**4**) and it showed anti-proliferative activity toward ovarian adenoma cell lines [23]. In the same vein, the symmetric bis-isatin derivative (**7**, Fig 2) displayed *in vitro* anti-proliferative activity against the HepG2 cell line with IC_50_ value about 4.23 mM [21].

Microsphere carrier systems made from the naturally occurring biodegradable polymers have attracted considerable attention for several years in the field of sustained drug delivery. These carrier systems can precisely control the release rates and target drugs to a specific body site with an enormous impact in formulation and development of novel drug delivery systems [24]. Biodegradable polyesters such as copolymers of lactic and glycolic acid (PLGA) are attractive biomaterials for the encapsulation of pharmaceuticals and bioactive compounds. In this case, encapsulated drugs are not as readily available to biological systems as when they are in solution because the release of the drug will occur only after the onset of polymeric degradation [25].

In this context, certain potential functionalized Schiff bases of isatin **4a-o** were designed and synthesized to evaluate their *in vitro* anti-proliferative activity against a panel of human cancer cell lines. In addition, the non-symmetric bis-isatins **7a-e** were also prepared and evaluated as new anti-proliferative agents. The compound exhibiting the best pharmacological activity was subjected to deeper pharmacological investigations in order to gain insight into its pharmacological profile as well as its possible mechanism of action. Moreover, this compound was encapsulated into PLGA microspheres in order to evaluate the influence of microencapsulation on its *in vitro* anti-proliferative activity.

## Experimental

### General

Melting points were measured with a Stuart melting point apparatus and were uncorrected. Infrared (IR) spectra were recorded as KBr disks using an FT-IR Spectrum BX apparatus(Perkin Elmer, CT, USA). NMR spectra were recorded on a Bruker NMR spectrometer (Bruker, Reinstetten, Germany).^1^H spectra were run at 500 MHz and ^13^C spectra were run at 125 MHz in deuterated dimethyl sulfoxide (DMSO-*d*_*6*_). Chemical shifts are expressed in values (ppm) using the solvent peak as an internal standard. All coupling constant (*J*) values are given in Hz. The abbreviations used are as follows: s, singlet; d, doublet; m, multiplet. Mass spectra were recorded using an Agilent Quadrupole 6120 LC/MS with an ESI (electrospray ionization) source (Agilent Technologies, Palo Alto, CA, USA). Elemental analyses were carried out at the regional center for microbiology and biotechnology, Al-Azhar University, Cairo, Egypt. Analytical thin layer chromatography (TLC) on silica gel plates containing a UV indicator was employed routinely to follow the course of reactions and to check the purity of the products. All reagents and solvents were purified and dried by standard techniques. Compounds **4a** [26], **4f** [27], **4i** [28] and **6a, b** [29] have been previously reported. The microspheres were prepared with poly (D, L-lactide co-glycolide) PLGA (50:50, mol. wt 30,000–60,000), which was purchased from Sigma-Aldrich (St. Louis, USA). The emulsifier, low molecular weight polyvinyl alcohol (PVA) was obtained from Alfa Aesar (Karlsruhe, Germany). Dichloromethane (DCM) was purchased from Avonchem (United Kingdom). Dimethyl sulfoxide (DMSO) was obtained from Loba Chemie (Mumbai, India). All ingredients used were of analytical grade. All cell lines have been purchased from the American Type Culture Collection (ATCC).

### Chemistry

#### 3-Hydrazonoindolin-2-ones2a-c

To a stirred solution of indoline-2,3-diones 1a-c (10 mmol) in methanol (20 mL), 99% hydrazine hydrate (2.5 mL, 50 mmol) was added. The reaction mixture was refluxed for one hour. The precipitate was filtered, washed with methanol, dried and re-crystallized from glacial acetic acid to afford intermediates 2a-c [30].

#### General procedure for the synthesis of hydrazones 4a-o

A catalytic amount of glacial acetic acid was added to equimolar quantities of the hydrazones **2a-c** (1 mmol) and benzaldehydes **3a-e** (1 mmol) in ethanol (10 mL).The reaction mixture was refluxed for four hours and then cooled to room temperature. The obtained precipitate was collected by filtration, dried and re-crystallized from ethanol/dioxane mixture (5:1) to afford compounds **4a-o** with 67–83% yields.

3-((4-Methoxybenzylidene)hydrazono)indolin-2-one (**4a**). Orange powder (yield 73%), m.p. 277–279°C; IR (KBr, *ν* cm^-1^): 3405 (NH), 1734 (C = O), 1607 (C = N); ^1^H NMR (DMSO-*d*_6_) *ppm*: 3.87 (s, 3H, OCH_3_), 6.90 (d, 1H, H-7 isatin, *J* = 7.5 Hz), 7.05 (t, 1H, H-5 isatin, *J* = 7.5 Hz), 7.14 (d, 2H, H-3 and H-5 of 4-OCH_3_-C_6_H_4_, *J* = 8.0 Hz), 7.39 (t, 1H, H-6 isatin, *J* = 7.5 Hz), 7.96 (d, 2H, H-2 and H-6 of 4-OCH_3_-C_6_H_4_, *J* = 8.0 Hz), 8.04 (d, 1H, H-4 isatin, *J* = 7.5 Hz), 8.64 (s, 1H, -CH =), 10.84 (s, 1H, NH_indolic_); ^13^C NMR (DMSO-*d*_6_) *ppm*: 56.0 (OCH_3_), 111.2, 112.4, 115.3, 117.1, 122.8, 126.6, 127.3, 129.4, 131.5, 133.9, 145.3, 162.4, 163.2 (Ar-CH Ar-C and C = O); MS (ESI) *m/z*: 280.1 [M+1]^+^; Anal. Calcd. for C_16_H_13_N_3_O_2_: C, 68.81; H, 4.69; N, 15.05; Found: C, 69.03; H, 4.64; N, 14.94.

3-((3-Hydroxy-4-methoxybenzylidene)hydrazono)indolin-2-one (**4b**). Orange powder (yield 71%), m.p. 273–275°C; IR (KBr, *ν* cm^-1^): 3282 (NH), 1720 (C = O), 1615 (C = N); ^1^H NMR (DMSO-*d*_6_) *ppm*: 3.87 (s, 3H, OCH_3_), 6.90–7.11 (m, 3H, Ar-H), 7.22–7.53 (m, 4H, Ar-H), 8.52 (s, 1H, -CH =), 9.31 (s, 1H, OH), 11.00 (s, 1H, NH_indolic_); ^13^C NMR (DMSO-*d*_6_) *ppm*: 56.1 (OCH_3_), 112.7, 113.8, 116.3, 119.4, 122.6, 123.0, 124.8, 127.6, 129.2, 133.3, 134.9, 145.1, 147.3, 162.4, 163.2 (Ar-CH, Ar-C and C = O); MS (ESI) *m/z*: 296.1 [M+1]^+^; Anal. Calcd. for C_16_H_13_N_3_O_3_: C, 65.08; H, 4.44; N, 14.23; Found: C, 65.24; H, 4.39; N, 14.35.

3-((4-Hydroxy-3-methoxybenzylidene)hydrazono)indolin-2-one (**4c**). Red powder (yield 67%), m.p. 269–270°C; IR (KBr, *ν* cm^-1^):3448 (NH), 1718 (C = O), 1618 (C = N); ^1^H NMR (DMSO-*d*_6_) *ppm*: 3.88 (s, 3H, OCH_3_), 6.91 (d, 1H, H-7 isatin, *J* = 7.5 Hz), 6.96 (d, 1H, H-5 of 4-OH-3-OCH_3_-C_6_H_3_, *J* = 8.0 Hz), 7.02 (t, 1H, H-5 isatin, *J* = 7.5 Hz), 7.40 (t, 1H, H-6 isatin, *J* = 7.5 Hz), 7.46 (d, 1H, H-6 of 4-OH-3-OCH_3_-C_6_H_3_, *J* = 8.5 Hz), 8.02 (d, 1H, H-4 isatin, *J* = 8.0 Hz), 7.59 (s, 1H, H-2 of 4-OH-3-OCH_3_-C_6_H_3_), 8.70 (s, 1H, -CH =), 10.14 (s, 1H, OH), 10.98 (s, 1H, NH_indolic_); ^13^C NMR (DMSO-*d*_6_) *ppm*: 56.1 (OCH_3_), 111.2, 112.1, 116.4, 117.2, 122.8, 124.7, 125.3, 127.5, 129.3, 133.8, 139.1, 145.2, 148.6, 151.0, 165.3 (C = O); MS (ESI) *m/z*: 296.1 [M+1]^+^; Anal. Calcd. for C_16_H_13_N_3_O_3_: C, 65.08; H, 4.44; N, 14.23; Found: C, 64.86; H, 4.39; N, 14.08.

3-((3,4-Dimethoxybenzylidene)hydrazono)indolin-2-one (**4d**). Orange powder (yield 79%), m.p. 276–278°C; IR (KBr, *ν* cm^-1^): 3281 (NH), 1721 (C = O), 1616 (C = N); ^1^H NMR (DMSO-*d*_6_) *ppm*: 3.87 (s, 3H, OCH_3_), 3.88 (s, 3H, OCH_3_), 6.90 (d, 1H, H-7 isatin, *J* = 7.5 Hz), 7.04 (t, 1H, H-5 isatin, *J* = 7.5 Hz), 7.16 (d, 1H, H-5 of 3,4-(OCH_3_)_2_-C_6_H_3_, *J* = 8.0 Hz), 7.39 (t, 1H, H-6 isatin, *J* = 7.5 Hz), 7.56–7.58 (m, 2H, H-2 and H-6 of 3,4-(OCH_3_)_2_-C_6_H_3_), 8.01 (d, 1H, H-4 isatin, *J* = 8.0 Hz), 8.58 (s, 1H, -CH =), 10.84 (s, 1H, NH_indolic_); ^13^C NMR (DMSO-*d*_6_) *ppm*: 55.9 (OCH_3_), 56.1 (OCH_3_), 109.6, 111.6, 111.9, 116.3, 123.0, 123.9, 127.2, 128.7, 134.9, 145.2, 145.7, 149.5, 152.1, 161.2, 163.9 (Ar-CH, Ar-C and C = O); MS (ESI) *m/z*: 310.1 [M+1]^+^; Anal. Calcd. for C_17_H_15_N_3_O_3_: C, 66.01; H, 4.89; N, 13.58; Found: C, 65.79; H, 4.92; N, 13.72.

3-((3,4,5-Trimethoxybenzylidene)hydrazono)indolin-2-one (**4e**). Red powder (yield 80%), m.p. 240–241°C; IR (KBr, *ν* cm^-1^): 3280 (NH), 1721 (C = O), 1616 (C = N); ^1^H NMR (DMSO-*d*_6_) *ppm*: 3.74 (s, 3H, OCH_3_), 3.85 (s, 6H, 2 OCH_3_), 6.92 (d, 1H, H-7 isatin, *J* = 7.5 Hz), 7.01 (t, 1H, H-5 isatin, *J* = 7.5 Hz), 7.23 (s, 2H, H-2 and H-6 of 3,4,5-(OCH_3_)_3_-C_6_H_2_), 7.41 (t, 1H, H-6 isatin, *J* = 7.5 Hz), 7.51 (d, 1H, H-4 isatin, *J* = 7.5 Hz), 8.67 (s, 1H, -CH =), 11.00 (s, 1H, NH_indolic_); ^13^C NMR (DMSO-*d*_6_) *ppm*: 56.4 (OCH_3_), 61.1 (OCH_3_), 106.0, 111.6, 116.2, 123.0, 128.7, 129.7, 134.9, 140.6, 145.2, 145.7, 153.6, 161.8, 163.9 (C = O); MS (ESI) *m/z*: 340.1 [M+1]^+^; Anal. Calcd. for C_18_H_17_N_3_O_4_: C, 63.71; H, 5.05; N, 12.38; Found: C, 63.95; H, 5.01; N, 12.29.

5-Chloro-3-((4-methoxybenzylidene)hydrazono)indolin-2-one (**4f**). Orange powder (yield 75%), m.p. 282–284°C; IR (KBr, *ν* cm^-1^): 3421 (NH), 1725 (C = O), 1618 (C = N); ^1^H NMR (DMSO-*d*_6_) *ppm*: 3.88 (s, 3H, OCH_3_), 6.93 (d, 1H, H-6 isatin, *J* = 8.0 Hz), 7.17 (d, 2H, H-3 and H-5 of 4-OCH_3_-C_6_H_4_, *J* = 8.5 Hz), 7.46 (d, 1H, H-7 isatin, *J* = 8.5 Hz), 7.94 (d, 2H, H-2 and H-6 of 4-OCH_3_-C_6_H_4_, *J* = 8.5 Hz), 8.01 (s, 1H, H-4 isatin), 8.70 (s, 1H, -CH =), 10.98 (s, 1H, NH_indolic_); ^13^C NMR (DMSO-*d*_6_) *ppm*: 56.1 (OCH_3_), 112.8, 115.4, 118.2, 126.3, 126.3, 128.5, 131.6, 133.3, 144.1, 150.5, 163.4, 163.9, 164.9 (Ar-CH, Ar-C and C = O); MS (ESI) *m/z*: 314.1 [M+1]^+^; Anal. Calcd. for C_16_H_12_ClN_3_O_2_: C, 61.25; H, 3.86; N, 13.39; Found: C, 61.08; H, 3.90; N, 13.53.

5-Chloro-3-((3-hydroxy-4-methoxybenzylidene)hydrazono)indolin-2-one(**4g**). Orange powder (yield 72%), m.p. 278–280°C; IR (KBr, *ν* cm^-1^): 3364 (NH), 1725 (C = O), 1616 (C = N); ^1^H NMR (DMSO-*d*_6_) *ppm*: 3.88 (s, 3H, OCH_3_), 6.93 (d, 1H, H-6 isatin, *J* = 8.0 Hz), 7.13 (d, 1H, H-5 of 3-OH-4-OCH_3_-C_6_H_3_, *J* = 8.5 Hz), 7.39 (d, 1H, H-7 isatin, *J* = 8.0 Hz), 7.46–7.48 (m, 2H, H-2 and H-6 of 3-OH-4-OCH_3_-C_6_H_3_), 8.02 (s, 1H, H-4 isatin), 8.61 (s, 1H, -CH =), 9.61 (s, 1H, OH), 10.97 (s, 1H, NH_indolic_); ^13^C NMR (DMSO-*d*_6_) *ppm*: 56.2 (OCH_3_), 112.6, 112.8, 114.2, 118.3, 124.0, 126.3, 126.5, 128.4, 133.3, 144.0, 147.6, 150.3, 152.6, 164.4, 164.9 (Ar-CH, Ar-C and C = O); MS (ESI) *m/z*: 330.1 [M+1]^+^; Anal. Calcd. for C_16_H_12_ClN_3_O_3_: C, 58.28; H, 3.67; N, 12.74; Found: C, 58.41; H, 3.64; N, 12.67.

5-Chloro-3-((4-hydroxy-3-methoxybenzylidene)hydrazono)indolin-2-one (**4h**). Orange powder (yield 70%), m.p. 269–271°C; IR (KBr, *ν* cm^-1^): 3263 (NH), 1725 (C = O), 1615 (C = N); ^1^H NMR (DMSO-*d*_6_) *ppm*: 3.90 (s, 3H, OCH_3_), 6.92 (d, 1H, H-6 isatin, *J* = 8.0 Hz), 6.97 (d, 1H, H-5 of 4-OH-3-OCH_3_-C_6_H_3_, *J* = 8.5 Hz), 7.42–7.46 (m, 2H, H-6 of 4-OH-3-OCH_3_-C_6_H_3_ and H-7 isatin), 7.59 (s, 1H, H-2 of 4-OH-3-OCH_3_-C_6_H_3_), 8.14 (s, 1H, H-4 isatin), 8.66 (s, 1H, -CH =), 10.16 (s, 1H, OH), 10.96 (s, 1H, NH_indolic_); ^13^C NMR (DMSO-*d*_6_) *ppm*: 55.8 (OCH_3_), 111.2, 112.8, 116.4, 118.4, 125.2, 125.7, 126.2, 128.8, 133.1, 143.9, 148.7, 150.7, 152.2, 164.8, 165.1 (Ar-CH, Ar-C and C = O); MS (ESI) *m/z*: 330.1 [M+1]^+^; Anal. Calcd. for C_16_H_12_ClN_3_O_3_: C, 58.28; H, 3.67; N, 12.74; Found: C, 58.09; H, 3.70; N, 12.87.

5-Chloro-3-((3,4-dimethoxybenzylidene)hydrazono)indolin-2-one(**4i**). Red powder (yield 81%), m.p. 282–284°C; IR (KBr, *ν* cm^-1^): 3420(NH), 1744 (C = O), 1818 (C = N); ^1^H NMR (DMSO-*d*_6_) *ppm*: 3.88 (s, 6H, 2 OCH_3_), 6.93 (d, 1H, H-6 isatin, *J* = 8.0 Hz), 7.19 (d, 1H, H-5 of 3,4-(OCH_3_)_2_-C_6_H_3_, *J* = 8.5 Hz), 7.46 (d, 1H, H-7 isatin, *J* = 8.0 Hz), 7.54 (d, 1H, H-6 of 3,4-(OCH_3_)_2_-C_6_H_3_, *J* = 8.0 Hz), 7.61 (s, 1H, H-2 of 3,4-(OCH_3_)_2_-C_6_H_3_), 8.01 (s, 1H, H-4 isatin), 8.69 (s, 1H, -CH =), 10.99 (s, 1H, NH_indolic_); ^13^C NMR (DMSO-*d*_6_) *ppm*: 55.7 (OCH_3_), 56.3 (OCH_3_), 110.0, 112.3, 112.8, 118.3, 125.4, 126.2, 126.4, 128.8, 133.3, 144.0, 149.6, 150.9, 153.4, 164.0, 164.9 (C = O); MS (ESI) *m/z*: 343.9 [M+1]^+^; Anal. Calcd. for C_17_H_14_ClN_3_O_3_: C, 59.40; H, 4.11; N, 12.22; Found: C, 59.61; H, 4.07; N, 12.34.

5-Chloro-3-((3,4,5-trimethoxybenzylidene)hydrazono)indolin-2-one (**4j**). Red powder (yield 77%), m.p. 245–247°C; IR (KBr, *ν* cm^-1^): 3315 (NH), 1734 (C = O), 1617 (C = N); ^1^H NMR (DMSO-*d*_6_) *ppm*: 3.78 (s, 3H, OCH_3_), 3.90 (s, 6H, 2 OCH_3_), 6.93 (d, 1H, H-6 isatin, *J* = 7.5 Hz), 7.34 (s, 2H, H-2 and H-6 of 3,4,5-(OCH_3_)_3_-C_6_H_2_), 7.46 (d, 1H, H-7 isatin, *J* = 7.0 Hz), 8.03 (s, 1H, H-4 isatin), 8.63 (s, 1H, -CH =), 11.01 (s, 1H, NH_indolic_); ^13^C NMR (DMSO-*d*_6_) *ppm*: 56.4 (OCH_3_), 60.8 (OCH_3_), 106.7, 112.9, 118.2, 126.3, 128.9, 129.1, 133.4, 141.7, 144.2, 151.4, 153.8, 162.6, 164.8 (Ar-CH, Ar-C and C = O); MS (ESI) *m/z*: 373.2 [M+1]^+^; Anal. Calcd. for C_18_H_16_ClN_3_O_4_: C, 57.84; H, 4.31; N, 11.24; Found: C, 58.07; H, 4.26; N, 11.31.

5-Bromo-3-((4-methoxybenzylidene)hydrazono)indolin-2-one (**4k**). Orange powder (yield 76%), m.p. 290–292°C; IR (KBr, *ν* cm^-1^): 3422 (NH), 1744 (C = O), 1616 (C = N); ^1^H NMR (DMSO-*d*_6_) *ppm*: 3.88 (s, 3H, OCH_3_), 6.88 (d, 1H, H-6 isatin, *J* = 8.0 Hz), 7.18 (d, 2H, H-3 and H-5 of 4-OCH_3_-C_6_H_4_, *J* = 8.5 Hz), 7.59 (d, 1H, H-7 isatin, *J* = 8.0 Hz), 7.93 (d, 2H, H-2 and H-6 of 4-OCH_3_-C_6_H_4_, *J* = 8.5 Hz), 8.16 (s, 1H, H-4 isatin), 8.69 (s, 1H, -CH =), 10.99 (s, 1H, NH_indolic_); ^13^C NMR (DMSO-*d*_6_) *ppm*: 56.1 (OCH_3_), 113.3, 113.9, 115.4, 118.7, 126.3, 131.3, 131.6, 136.1, 144.2, 150.4, 163.4, 163.9, 164.8 (C = O); MS (ESI) *m/z*: 358.4 [M+1]^+^; Anal. Calcd. for C_16_H_12_BrN_3_O_2_: C, 53.65; H, 3.38; N, 11.73; Found: C, 53.78; H, 3.36; N, 11.62.

5-Bromo-3-((3-hydroxy-4-methoxybenzylidene)hydrazono)indolin-2-one (**4l**). Orange powder (yield 74%), m.p. 278–280°C; IR (KBr, *ν* cm^-1^): 3361 (NH), 1725 (C = O), 1611 (C = N); ^1^H NMR (DMSO-*d*_6_) *ppm*: 3.88 (s, 3H, OCH_3_), 6.89 (d, 1H, H-6 isatin, *J* = 8.0 Hz), 7.14 (d, 1H, H-5 of 3-OH-4-OCH_3_-C_6_H_3_, *J* = 8.5 Hz), 7.51 (d, 1H, H-7 isatin, *J* = 8.0 Hz), 7.45–7.47 (m, 2H, H-2 and H-6 of 3-OH-4-OCH_3_-C_6_H_3_), 8.25 (s, 1H, H-4 isatin), 8.69 (s, 1H, -CH =), 9.59 (s, 1H, OH), 10.99 (s, 1H, NH_indolic_); ^13^C NMR (DMSO-*d*_6_) *ppm*: 56.3 (OCH_3_), 112.6, 113.3, 113.9, 114.4, 118.7, 123.9, 126.5, 131.2, 136.1, 144.4, 147.6, 150.2, 152.6, 164.2, 164.8 (Ar-CH, Ar-C and C = O); MS (ESI) *m/z*: 374.1 [M+1]^+^; Anal. Calcd. for C_16_H_12_BrN_3_O_3_: C, 51.36; H, 3.23; N, 11.23; Found: C, 51.52; H, 3.26; N, 11.08.

5-Bromo-3-((4-hydroxy-3-methoxybenzylidene)hydrazono)indolin-2-one (**4m**). Red powder (yield 72%), m.p. 285–287°C; IR (KBr, *ν* cm^-1^): 3420 (NH), 1745 (C = O), 1609 (C = N); ^1^H NMR (DMSO-*d*_6_) *ppm*: 3.92 (s, 3H, OCH_3_), 6.87 (d, 1H, H-6 isatin, *J* = 8.0 Hz), 6.97 (d, 1H, H-5 of 4-OH-3-OCH_3_-C_6_H_3_, *J* = 8.0 Hz), 7.41 (d, 1H, H-7 isatin, *J* = 8.0 Hz), 7.57 (d, 1H, H-6 of 4-OH-3-OCH_3_-C_6_H_3_, *J* = 8.5 Hz), 7.60 (s, 1H, H-2 of 4-OH-3-OCH_3_-C_6_H_3_), 8.31 (s, 1H, H-4 isatin), 8.66 (s, 1H, -CH =), 10.17 (s, 1H, OH), 10.97 (s, 1H, NH_indolic_); ^13^C NMR (DMSO-*d*_6_) *ppm*: 55.8 (OCH_3_), 110.8, 113.2, 113.9, 116.3, 118.9, 125.3, 125.9, 131.7, 135.8, 144.3, 148.7, 150.7, 152.2, 164.8, 164.9 (Ar-CH, Ar-C and C = O); MS (ESI) *m/z*: 374.1 [M+1]^+^; Anal. Calcd. for C_16_H_12_BrN_3_O_3_: C, 51.36; H, 3.23; N, 11.23; Found: C, 51.09; H, 3.29; N, 11.35.

5-Bromo-3-((3,4-dimethoxybenzylidene)hydrazono)indolin-2-one (**4n**). Red powder (yield 83%), m.p. 280–282°C; IR (KBr, *ν* cm^-1^): 3421 (NH), 1744 (C = O), 1618 (C = N); ^1^H NMR (DMSO-*d*_6_) *ppm*: 3.88 (s, 6H, 2 OCH_3_), 6.88 (d, 1H, H-6 isatin, *J* = 8.5 Hz), 7.18 (d, 1H, H-5 of 3,4-(OCH_3_)_2_-C_6_H_3_, *J* = 8.5 Hz), 7.52 (d, 1H, H-7 isatin, *J* = 8.5 Hz), 7.58 (d, 1H, H-6 of 3,4-(OCH_3_)_2_-C_6_H_3_, *J* = 8.5 Hz), 7.60 (s, 1H, H-2 of 3,4-(OCH_3_)_2_-C_6_H_3_), 8.27 (s, 1H, H-4 isatin), 8.69 (s, 1H, -CH =), 10.99 (s, 1H, NH_indolic_); ^13^C NMR (DMSO-*d*_6_) *ppm*: 55.8 (OCH_3_), 56.3 (OCH_3_), 111.4, 113.3, 119.3,123.6, 126.5, 129.4, 132.3, 136.9, 141.4, 144.3, 147.6, 149.7, 151.1, 162.3, 164.0(Ar-CH, Ar-C and C = O); MS (ESI) *m/z*: 388.2[M+1]^+^; Anal. Calcd. for C_17_H_14_BrN_3_O_3_: C, 52.60; H, 3.64; N, 10.82; Found: C, 52.80; H, 3.61; N, 10.71.

5-Bromo-3-((3,4,5-trimethoxybenzylidene)hydrazono)indolin-2-one (**4o**). Red powder (yield 81%), m.p. 261–263°C; IR (KBr, *ν* cm^-1^): 3413 (NH), 1737 (C = O), 1612 (C = N); ^1^H NMR (DMSO-*d*_6_) *ppm*: 3.78 (s, 3H, OCH_3_), 3.90 (s, 6H, 2 OCH_3_), 6.88 (d, 1H, H-6 isatin, *J* = 8.0 Hz), 7.34 (s, 2H, H-2 and H-6 of 3,4,5-(OCH_3_)_3_-C_6_H_2_), 7.58 (d, 1H, H-7 isatin, *J* = 8.0 Hz), 8.21 (s, 1H, H-4 isatin), 8.64 (s, 1H, -CH =), 11.01 (s, 1H, NH_indolic_); ^13^C NMR (DMSO-*d*_6_) *ppm*: 56.4 (OCH_3_), 60.8 (OCH_3_), 106.7, 113.36, 113.9, 118.7, 129.1, 131.9, 136.2, 141.7, 144.5, 151.1, 153.8, 162.8, 164.7 (Ar-CH, Ar-C and C = O); MS (ESI) *m/z*: 418.2 [M+1]^+^; Anal. Calcd. for C_18_H_16_BrN_3_O_4_: C, 51.69; H, 3.86; N, 10.05; Found: C, 51.84; H, 3.82; N, 9.91.

#### General procedure for the synthesis of target indolin-2-ones7a-e

A mixture of intermediates 6a, b (1 mmol) and the appropriate 3-hydrazonoindolin-2-one2a-d (1 mmol) in ethyl alcohol and catalytic amount of glacial acetic acid was heated under reflux for six hours, filtered while hot and the precipitate was washed with ethanol. The solid product was collected and re-crystallized from an ethanol/DMF mixture (3:1) to furnish compounds 7a-e with 67–79% yield.

5-Fluoro-3-((1-methyl-2-oxoindolin-3-ylidene)hydrazono)indolin-2-one (**7a**). Red powder (yield 72%), m.p. 295–297°C; IR (KBr, *ν* cm^-1^): 3409 (NH), 1737, 1702 (2 C = O), 1611 (C = N); ^1^H NMR (DMSO-*d*_6_) *ppm*: 3.22 (s, 3H, CH_3_), 6.92–6.94 (m, 1H, Ar-H), 7.10–7.14 (m, 2H, Ar-H), 7.31–7.33 (m, 2H, Ar-H), 7.52–7.55 (m, 2H, Ar-H), 11.03 (s, 1H, NH_indolic_); ^13^C NMR (DMSO-*d*_6_) *ppm*: 27.2 (CH_3_), 110.3, 112.6, 115.7, 116.6, 121.3, 121.4, 123.5, 128.6, 134.9, 142.1, 145.3, 146.9, 157.4, 158.7, 162.6 (C = O), 163.9 (C = O); MS *m/z*: 323.1 [M+1]^+^; Anal. Calcd. for C_17_H_11_FN_4_O_2_: C, 63.35; H, 3.44; N, 17.38; Found: C, 63.53; H, 3.40; N, 17.51.

5-Chloro-3-((methyl-2-oxoindolin-3-ylidene)hydrazono)indolin-2-one (**7b**). Red powder (yield 74%), m.p. 269–271°C; IR (KBr, *ν* cm^-1^): 3409 (NH), 1735, 1707 (2C = O),1638 (C = N); ^1^H NMR (DMSO-*d*_6_) *ppm*: 3.22 (s, 3H, CH_3_), 6.94 (d, 1H, Ar-H, *J* = 8.5 Hz), 7.08–7.14 (m, 2H, Ar-H), 7.48–7.56 (m, 4H, Ar-H), 11.14 (s, 1H, NH_indolic_);); ^13^C NMR (DMSO-*d*_6_) *ppm*: 26.7 (CH_3_), 110.3, 113.1, 115.7, 117.5, 123.5, 125.3, 128.1, 128.7, 134.3, 135.0, 137.6, 144.5, 145.3, 147.5, 162.4, 163.7 (Ar-CH, Ar-C and C = O); MS (ESI) *m/z*: 339.0 [M+1]^+^; Anal. Calcd. for C_17_H_11_ClN_4_O_2_: C, 60.28; H, 3.27; N, 16.54; Found: C, 60.49; H, 3.31; N, 16.41.

1-Benzyl-3-((oxoindolin-3-ylidene)hydrazono)indolin-2-one (**7c**). Red powder (yield 70%), m.p. 255–257°C; IR (KBr, *ν* cm^-1^): 3422 (NH), 1718 (C = O), 1608 (C = N); ^1^H NMR (DMSO-*d*_6_) *ppm*: 5.01 (s, 2H, CH_2_), 6.93 (d, 1H, Ar-H, *J* = 8.0 Hz), 6.97–7.12 (m, 3H, Ar-H), 7.28–7.49 (m, 9H, Ar-H), 11.02 (s, 1H, NH_indolic_); ^13^C NMR (DMSO-*d*_6_) *ppm*: 43.5 (CH_2_), 110.9, 111.6, 115.9, 116.2, 123.1, 123.7, 127.8, 128.1, 128.9,129.2, 134.7, 134.8, 135.1, 136.3,144.6, 144.8, 145.4, 145.8, 162.8 (C = O), 164.0 (C = O); MS (ESI) *m/z*: 381.1[M+1]^+^; Anal. Calcd. for C_23_H_16_N_4_O_2_: C, 72.62; H, 4.24; N, 14.73; Found: C, 72.39; H, 4.30; N, 14.87.

1-Benzyl-3-((5-chloro-2-oxoindolin-3-ylidene)hydrazono)indolin-2-one (**7d**). Red powder (yield 75%), m.p. 258–260°C; IR (KBr, *ν* cm^-1^): 3411 (NH), 1735 (C = O), 1603 (C = N); ^1^H NMR (DMSO-*d*_6_) *ppm*: 5.01 (s, 2H, CH_2_), 6.95 (d, 1H, Ar-H, *J* = 8.5 Hz), 7.01 (d, 1H, Ar-H, *J* = 8.0 Hz), 7.08 (t, 1H, Ar-H, *J* = 8.0 Hz), 7.29–7.60 (m, 9H, Ar-H), 11.15 (s, 1H, NH_indolic_); ^13^C NMR (DMSO-*d*_6_) *ppm*: 43.4 (CH_2_), 111.5, 111.9, 117.4, 119.5, 123.8, 124.9, 125.2, 126.8, 127.7, 127.8, 128.1, 129.1, 129.2, 129.3, 136.0, 138.4, 151.6, 157.4, 163.1 (C = O), 165.8 (C = O); MS *m/z*: 415.1[M+1]^+^; Anal. Calcd. for C_23_H_15_ClN_4_O_2_: C, 66.59; H, 3.64; N, 13.51; Found: C, 66.72; H, 3.61; N, 13.62.

1-Benzyl-3-((5-bromo-2-oxoindolin-3-ylidene)hydrazono)indolin-2-on**e** (**7e**). Red powder (yield 79%), m.p. 265–267°C; IR (KBr, *ν* cm^-1^): 3421 (NH), 1734 (C = O), 1602 (C = N); ^1^H NMR (DMSO-*d*_6_) *ppm*: 5.01 (s, 2H, CH_2_), 6.89 (d, 1H, Ar-H, *J* = 8.0 Hz), 7.00 (d, 1H, Ar-H, *J* = 8.0 Hz), 7.08 (t, 1H, Ar-H, *J* = 7.5 Hz), 7.27–7.45 (m, 6H, Ar-H), 7.59–7.70 (m, 3H, Ar-H), 11.15 (s, 1H, NH_indolic_)[31]; ^13^C NMR (DMSO-*d*_6_) *ppm*: 43.5 (CH_2_), 110.9, 113.6, 114.2, 115.9, 117.9, 123.7, 127.8, 128.1, 128.9, 129.2, 130.9, 134.9, 136.3, 137.2, 144.9, 145.6, 145.9, 146.3, 162.8, 163.6 (Ar-CH, Ar-C and C = O); MS (ESI) *m/z*: 459.0[M+1]^+^; Anal. Calcd. for C_23_H_15_BrN_4_O_2_: C, 60.15; H, 3.29; N, 12.20; Found: C, 59.89; H, 3.33; N, 12.28.

### Pharmacological investigations

The detailed experimental procedures for pharmacological evaluation of the synthesized compounds were provided as supplementary materials.

### Microspheres

#### Preparation of 7e -loaded PLGA microspheres

PLGA microspheres were prepared by a modified emulsion-solvent diffusion method as described previously [32]. Briefly, the DCM (3 mL) phase containing PLGA was poured into the DMSO (4 mL) phase containing compound 7e to obtain a 7e-polymer ratio of 1:10. The resulting organic phase was added dropwise with the help of a syringe into an aqueous phase (25 mL) containing 1% (w/v) PVA using magnetic stirrer at 300 rpm and then homogenized at 13,500 rpm at room temperature for 3 min. The resulting emulsion was then transferred into water (25 mL) at room temperature and stirred at 200 rpm with a magnetic stirrer overnight until the solvent was evaporated. The resulting PLGA microspheres were recovered by centrifugation at 15000 rpm using a 3–30 K centrifuge (Sigma, Germany) for 15 min at 4°C, washed three times with distilled water (50 mL) to remove the residual PVA, re-suspended in distilled water (15 mL) and then lyophilized in a Telstar freeze-dryer (Terrassa, Spain) at -50°C with a pressure below 1 mbar for 24 h. Plain microspheres were prepared in a similar manner except for the absence of compound 7e in the DMSO phase.

#### Microsphere characterization

*Microsphere yield*: The percentage yield of microspheres was calculated using the weight in mg of the final product after drying by lyophilization with respect to the initial total weight in mg of the drug and the polymer used for the preparation in the emulsifying process according to Eq (1) [32, 33]. The equation below shows the percentage yield calculation.

$$\%\;Yield=\frac{Weightoftheobtsainedmicrospheres}{Weightofthepolymer+weightofthedrug}\times 100$$ (1)

*Encapsulation efficiency and compound* ***7e****loading*: 10 mg of quantitatively weighed microspheres were dissolved in DMSO (2 mL) and then sonicated for 15 min, then 0.1N HCl (5 mL) was added to precipitate the polymer. The solution was centrifuged at 10,000 rpm for 10 min. then the supernatant was diluted appropriately and analyzed for the content of **7e** spectrophotometrically (Biochrom Libra S22, UK) at λ _max_ of 318 nm to determine the **7e** content. The polymer did not interfere with the absorbance of**7e** at the specified wavelength. Theoretical **7e**-loading was determined by the entire **7e** present in the polymer solution in the microspheres. The **7e**-loading and entrapment efficiency were determined by formulas (2) and (3), respectively.

$$\%\;\mathrm{Drug}\;\mathrm{loading}=\frac{Amountofthedruginthemicrospheres}{Amountofthemicrospheres}\times 100$$ (2)

$$Entrapmentefficiency=\frac{Actualdrugloadinginmicrospheres}{Theoriticaldrugloading}\times 100$$ (3)

All of the measurements were conducted in triplicate.

*Determination of the particle size of microspheres*: Particle size of the prepared microspheres was measured using a dynamic light scattering particle size analyzer, model Zetasizer Nano-ZS, ZEN 3500, Malvern Instruments (Worcestershire, UK) at 25.0 ± 0.1°C[34, 35]. Lyophilized microspheres (1 mg) were re-suspended in distilled water (100 mL) and the particle size was recorded in triplicate.

*Morphological characterization of* ***7e****-loaded microspheres*: To investigate the morphological characterization of the prepared microspheres, they were mounted onto a double-sided adhesive tape attaching to an aluminum stub, then coated with gold and examined by scanning electron microscope (SEM, JEOL JSM-7001F, Japan) at 20 kV acceleration voltage with a secondary detector. The magnification was adjusted until a clear image of the surface of the microspheres appeared and the picture was then recorded [36].

*In vitro****7e****-release*: Studies on the *in vitro* release of **7e** from microspheres were carried out using the vial method as reported by Dubey *et al*. [34]. Compound **7e**-PLGA-microspheres containing 5 mg equivalent of compound **7e** were suspended in a vial containing phosphate buffer (10 mL, pH 7.4) with 0.3% Tween 80 to improve the solubility of **7e**. The vial was incubated in a water bath at 37 ± 0.5°C and vibrated horizontally at speed of 100 rpm. *In vitro* **7e** release was assessed by intermittently sampling the vial (2 mL) at predetermined time intervals (1, 2, 3, 4, 5, 6, 10, 12, 15, 18, 20 and 21 days), then replacing the volume with 2 mL of fresh phosphate buffer.

*Cell viability assay*: The cell viability assay was performed as described in the section of anti-proliferative activity in the supplementary materials.

## Results and discussion

### Chemistry

The synthetic strategy adopted for the preparation of the target derivatives is depicted in S3 and S4 Files. Indoline-2,3-diones **1a-c** were refluxed with 99% hydrazine hydrate in methanol to obtain the corresponding hydrazone derivatives **2a-c**.The reaction of hydrazones **2a-c** with different substituted benzaldehydes **3a-e** in ethanol in the presence of a catalytic amount of glacial acetic acid furnished the target derivatives **4a-o** with 67–83% yields (S3 File).

The IR spectra of compounds **4a-o** displayed absorption bands around 3400 cm^-1^ for the indolic NH group in addition to the absorption bands of carbonyl groups near 1720 cm^-1^ which is consistent with the previously reported ones for compounds **4a** [26] and **4f** [27]. Also, their ^1^H NMR spectra revealed a D_2_O-exchangeable signals in the region *δ* 10.84–11.01 ppm attributable to the NH protons of the indolin-2-one moieties, in addition to the signal of the methine protons (-CH = N-) in the region *δ* 8.55–8.70 ppm which is in accordance with those previously reported for compounds **4a** [26], **4f** [27] and **4i** [28]. Moreover, the ^13^C NMR spectra of compounds **4a-o** showed signals resonating around *δ* 163 ppm, characteristic of C = O carbons.

Direct methylation and benzylation of indoline-2,3-dione **1a** with dimethyl sulfate and benzyl bromide were carried out in the presence of sodium hydroxide or potassium carbonate to furnish intermediates **6a** and **6b**, respectively. The second group of the target indolin-2-ones **7a-e** was obtained in good yields (67–79%) through the reaction of the two intermediates **6a** and **6b** with the appropriate 3-hydrazonoindolin-2-one **2a-d** in refluxing ethyl alcohol using catalytic amount of glacial acetic acid (S4 File).

The IR spectra of the target compounds **7a-e** revealed the presence of the characteristic absorption bands due to the NH and carbonyl groups of indolin-2-one moieties. Moreover, the ^1^H NMR spectra of **7a-e** revealed the presence of the singlet indolic NH protons at a *δ* 11.02–11.15 ppm. Also, the signals of the aliphatic protons (N-CH_3_) of compounds **7a, b** were observed as singlet signals around *δ* 3.22 ppm, while the benzylic protons (-CH_2_) of **7c-e** appeared as singlet signals close to *δ* 5.01 ppm.

### Pharmacological investigations

#### Anti-proliferative activity

Twenty compounds, i.e. 4a-o and 7a-e, were analyzed for cancer cell growth inhibitory activity. These studies were carried out using cells derived from human lung, colon and breast tumors (A549, HT-29 and ZR-75 cells, respectively). This initial assessment of activity tested each compound in quadruplicate at a single concentration of 30 μM, if solubility permitted.

As indicated in Table 1, the test compounds **4a-j, 4l-n** and **7a-e** exhibited anti-proliferative activity against A549, HT-29 and ZR-75 cells with an average growth inhibition in the range from 3.5 to 97.7%, whereas compounds **4k** and **4o** stimulated the growth of the tested cell lines.

**Table 1:** Anti-proliferative (cell growth inhibitory activity at 30 μM concentration) activity of the target compounds 4a-o, 7a-e and sunitinib against HT-29, ZR-75 and A-549 cell lines.

Compound No.	HT-29	ZR-75	A-549	Average Growth Inhibition %
**4a**	19.5 ± 12.4	34.8 ± 23.7	27.1 ± 20.3	27.2
**4b**	81.9 ± 3.0	59.3 ± 11.1	66.3 ± 5.7	69.2
**4c**	74.9 ± 5.3	55.4 ± 23.4	54.2 ± 5.0	61.5
**4d**	81.7 ± 5.2	58.8 ± 4.3	82.5 ± 6.9	74.3
**4e**	81.9 ± 5.4	86.1 ± 3.8	93.6 ± 3.6	87.2
**4f**	6.1 ± 8.6	10.9 ± 14.0	8.4 ± 10.0	8.4
**4g**	33.5 ± 11.3	46.0 ± 6.4	44.2 ± 16.5	41.2
**4h**	41.6 ± 10.4	30.9 ± 9.5	37.3 ± 8.1	36.6
4i*	-3.6 ± 9.7	33.0 ± 13.0	4.9 ± 3.3	11.4
**4j**	6.9 ± 4.2	18.1 ± 21.2	1.7 ± 17.5	8.9
**4k**	-0.6 ± 7.2	-5.3 ± 18.1	-6.9 ± 9.4	-4.3
**4l**	45.9 ± 9.8	44.3 ± 18.4	46.6 ± 9.5	45.6
**4m**	42.1 ± 1.8	58.7 ± 16.0	83.7 ± 10.3	61.5
4n*	-2.0 ± 10.2	12.5 ± 21.9	0.1 ± 5.1	3.5
**4o**	-1.1 ± 9.0	3.1 ± 8.2	-3.4 ± 6.6	-0.5
**7a**	84.8 ± 3.6	78.6 ± 9.9	91.6 ± 4.8	85.0
**7b**	95.5 ± 3.4	95.5 ± 6.0	95.2 ± 2.8	95.4
**7c**	73.8 ± 4.4	92.1 ± 3.8	86.6 ± 9.9	84.2
**7d**	97.0 ± 1.8	97.8 ± 1.8	98.4 ± 0.6	97.7
**7e**	95.7 ± 4.2	96.1 ± 4.6	97.1 ± 3.3	96.3
Sunitinib	59.5 ± 2.3	90.7 ± 4.5	85.7 ± 2.7	78.7

**Fig 3:**
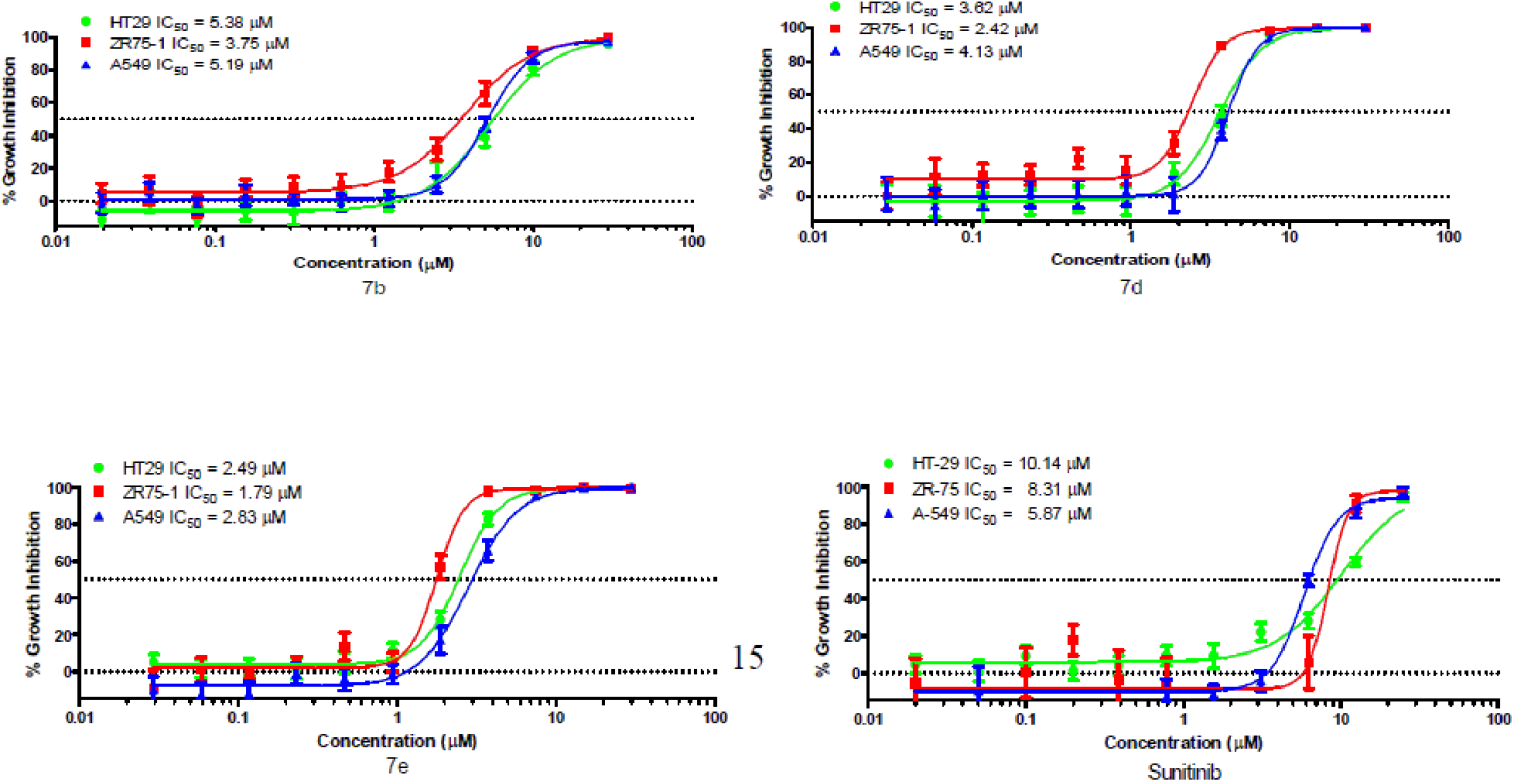
IC_50_ of the anti-proliferative activity of compounds 7b, 7d, 7e and sunitinib against HT-29, ZR-75 and A-549 cell lines.

Compounds **7b, 7d** and **7e** were the most active compounds, showing an average growth inhibition from 95.4 to 97.7%. Therefore, they were subjected to quantitative inhibitory concentration 50% (IC_50_) determination for their cell growth inhibitory activity towards the A549, HT-29 and ZR-75 cancer cell lines. The results are presented in Table 2 and Fig 3. Extra sum-of-squares F test was performed on dose response curves to determine if the IC_50_ values obtained in a cell line were significantly different for each compound tested.

**Table 2:** Inhibitory concentration 50% (IC_50_) of anti-proliferative activity of the selected compounds 7b, 7d, 7e and sunitinib against HT-29, ZR-75 and A-549 cell lines.

Compound No.	IC_50_ (μM)			Average IC_50_ (μM)
	HT-29	ZR-75	A-549	
**7b**	5.38^a^	3.75^a^	5.19^b^	4.77
**7d**	3.62^a^	2.42^a^	4.13^b^	3.39
**7e**	2.49^a^	1.79^a^	2.83^b^	2.37
Sunitinib	10.14^a^	8.31^a^	5.87^b^	8.11

^a^denotes those in which the *P*< 0.01

^b^denotes those in which the *P*< 0.05

Compound **7e** exhibited the best average IC_50_ value (2.37 μM), being nearly three-fold more potent than the positive control, sunitinib (average IC_50_ value = 8.11 μM). Therefore, compound **7e** was subjected to deeper pharmacological investigations in order to obtain insight into its pharmacological profile.

#### Apoptosis and caspase 3/7 activity

**Fig 4:**
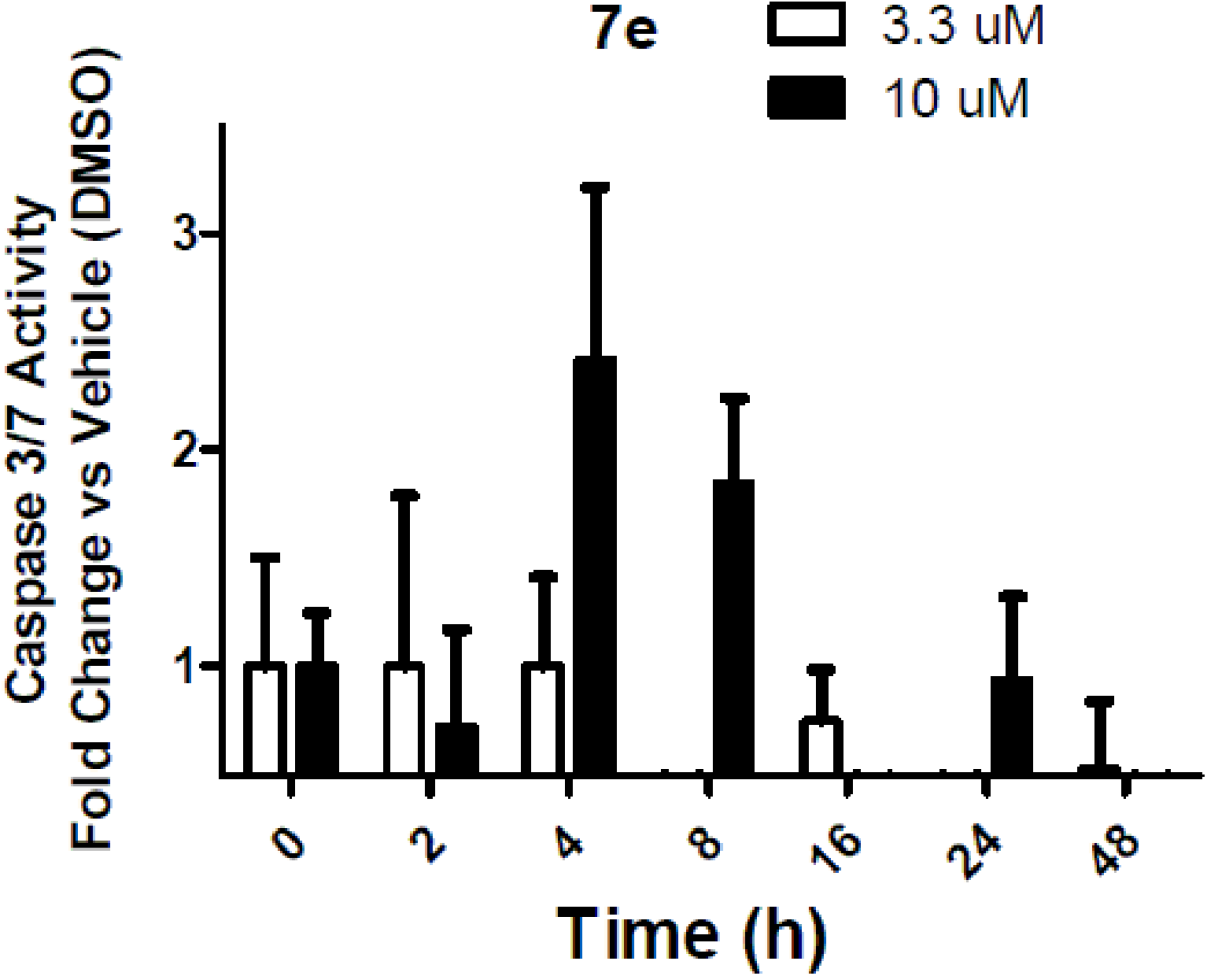
A-549 human lung cancer cells were treated with 3.3 and 10 μM 7e over a time course from 2–48 h. Caspase 3/7 activity was measured by a luminescence-based homogenous assay.

Compound 7e was analyzed for apoptosis inducing activity in cancer cells. These studies were carried out using cells derived from human lung (A549). This follow-up assessment of activity tested compounds in quadruplicate at concentrations equivalent to the IC_50_ value to inhibit growth and a concentration three-fold above the IC_50_ concentrations over a time course ranging from 2 to 48 h. As indicated in Fig 4, compound 7e at 10 μM increased caspase activity by 2.4- and 1.85-fold between 4 and 8 h of treatment, respectively, while the lower tested concentration had no effect on caspase 3/7 activity.

#### Cell cycle effects

Compound **7e** was analyzed for effects on various aspects of cell cycle progression in human cancer cells. These studies were carried out using cells derived from lung adenocarcinoma (A549). This follow-up assessment of activity tested compounds used immunofluorescent imaging of phosphorylated Rb protein and the total DNA content of each cell to determine the cell cycle phase. The ability of the test compounds to affect the cell cycle and Rb phosphorylation was tested over a range of concentrations from less than 100 nM to 50 μM.

**Fig 5:**
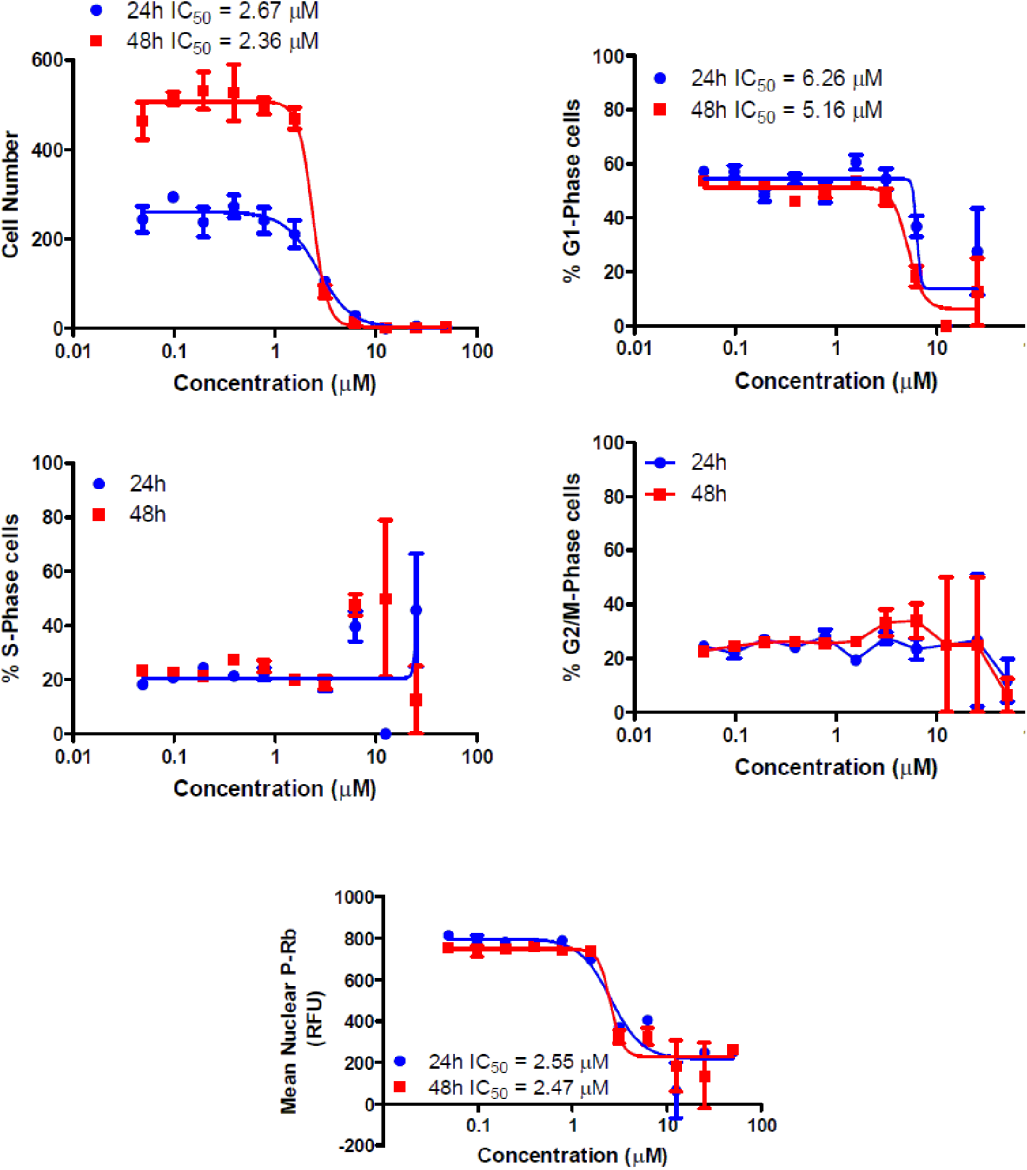
Dose-dependent changes in cell cycle. Distribution of cells within the cell cycle as well as total cell numbers were quantitated by fluorescent staining of nuclear DNA with DAPI. Phosphorylated Rb protein was detected by indirect immunofluorescence followed by automated image acquisition and analysis.

As indicated in Fig 5, compound **7e** had dose-dependent effects on the tested parameters. It caused a significant reduction in the total cell number after 24 h of treatment with an IC_50_ value = 2.67 μM and with an IC_50_ value = 2.36 μM after 48 h (Table 3), reflective of the dose-dependent growth inhibition observed in the homogenous assay.

**Table 3:** IC_50_ for reductions in the total cell number and cell cycle effects of compound 7e and sunitinib.

Compound No.	IC_50_ (μM) for reductions in the total cell number		IC_50_(μM)for reduction in Rb phosphorylation		Cell cycle effects
	24 h	48 h	24 h	48 h	
**7e**	2.67^a^	2.36	2.55^b^	2.47	G1 decreased, S-phase increased
Sunitinib	12.54^a^	3.48	3.16^b^	14.01	G1 decreased and G2/M-phases increased

^a^denotes those in which the *P*< 0.01

^b^denotes those in which the *P*< 0.05

In addition, compound **7e** caused a decrease in the percentage of cells in the G1 phase of the cell cycle with a corresponding increase in the S-phase. This suggests that part of the compound effects on growth may be attributable to a decreased rate of progression through the cell cycle and corresponding decreases in proliferation, similar to the positive control, sunitinib. Arrest in G2 may represent a checkpoint blockade, whereas mitotic arrest may, in some cases, lead to mitotic catastrophe and subsequent programmed death of cells with multiple or aberrant nuclei. The increase in S-phase cells without a concomitant G2/M phase fraction may indicate a very rapid onset of apoptosis in the case of compound **7e**.

As with other cell cycle parameters, levels of phosphorylated Rb protein were substantially reduced in a dose-dependent manner by the control and the test compound **7e**. After 24 h of treatment, the IC_50_ value was lower than the IC_50_ value for reductions in the cell number caused by the test compound **7e** (Table 3). This may support the hypothesis that the inhibition of cyclin dependent kinases by isatin compounds plays a role in their growth inhibitory activity. However, the correlation was less apparent at the 48 h time point.

**Fig 6:**
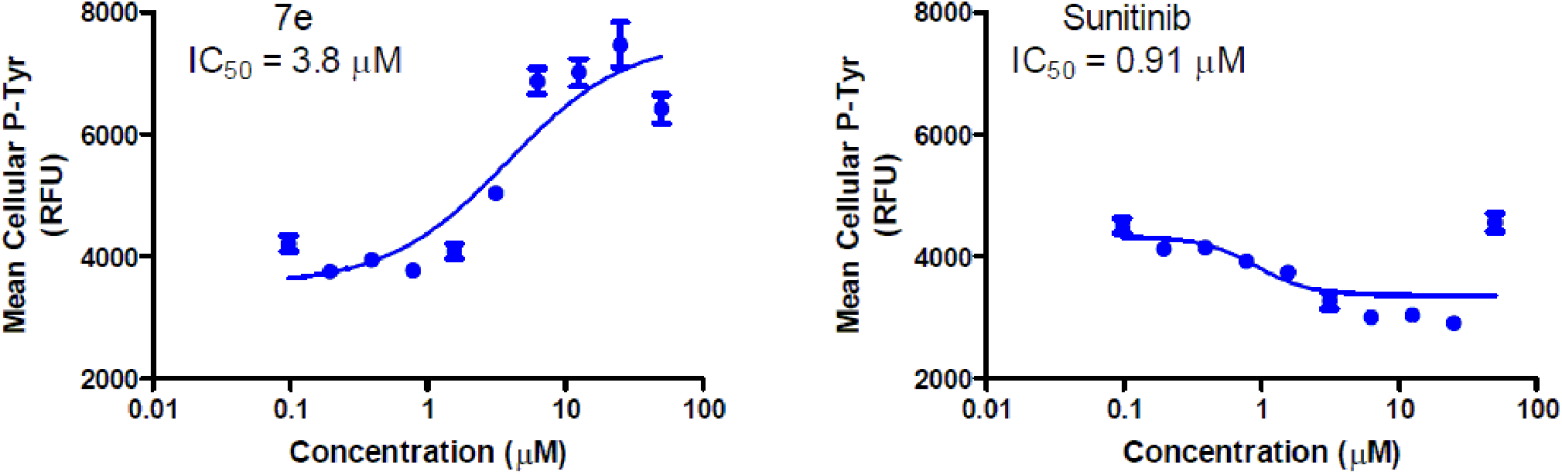
Dose-dependent changes in total levels of tyrosine phosphorylation. Cells were stimulated for 10 min with 10% fetal bovine serum. Phosphotyrosine levels were determined by indirect immunofluorescence followed by automated image acquisition and analysis.

Furthermore, compound **7e** was analyzed for its effect on total cellular levels of phosphorylated tyrosine (p-Tyr) residues in human cancer cells. These studies were carried out using cells derived from lung adenocarcinoma (A549) and immunofluorescent imaging. The ability of the test compound **7e** to affect acute serum stimulation of tyrosine phosphorylation was tested over a range of concentrations from less than 100 nM to 50 μM. Compound **7e** increased p-Tyr levels nearly two-fold with an apparent IC_50_ value of 3.8 μM, which is consistent with the growth inhibitory IC_50_ value of this compound. In contrast, the positive control compound sunitinib caused a modest dose-dependent decrease in the level of cellular tyrosine phosphorylation activity with an IC_50_ value of 0.9 μM, well below its growth inhibitory IC_50_value (Fig 6).

#### Selectivity

As an indicator of selectivity for tumor cells, compound **7e** was analyzed for cell growth inhibitory activity in three non-tumorigenic cell lines.IEC-6 cells derived from rat intestine exhibit morphologic and karyotypic features of normal intestinal epithelial cells [37].Cultures derived from human fibrocystic mammary tissue (MCF-10A) are non-tumorigenic and exhibit features of primary cultures of breast tissue, including dome formation [38].Fibroblasts derived from embryonic tissue from mice (Swiss 3t3 fibroblasts) are both non-tumorigenic and contact inhibited [39].For comparison, the A549 human non-small cell lung cancer (NSCLC) cell line was included. This assessment of the growth inhibitory activity of the compounds was tested in quadruplicate at a maximum concentration of 25 μM, followed by 10 serially diluted concentrations.

**Fig 7:**
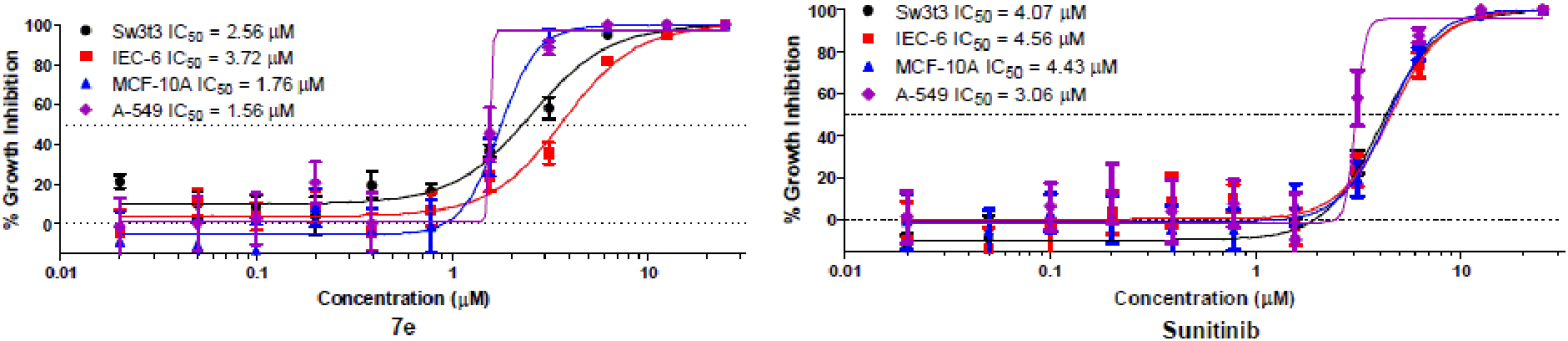
Tumor cell selectivity. Dose-dependent growth inhibition by **7e** or sunitinib in a NSCLC cell line (A-549) compared with three non-tumorigenic cell lines: Sw-3t3 (murine fibroblasts), IEC-6 (murine inestinal epithelial cells), or MCF-10A (human mammary epithelial cells).

As indicated in Fig 7 and Table 4, compound **7e** inhibited growth in both normal and tumor cell lines >50%. Compound **7e** displayed a selectivity value of 1.7 while the positive control sunitinib showed a selectivity value of 1.4 (the difference between the mean IC_50_ in non-tumor cell lines versus the NSCLC cells).

**Table 4:** Selectivity for compound 7e and sunitinib toward tumor and non-tumorigenic cell lines.

Compound No.	IC_50_ (μM)				Mean tumor selectivity
	Intestine IEC-6	Breast MCF-10A	Fibroblast Swiss 3t3	NSCLC A-549	
**7e**	3.72^a^	1.75	2.56 ^a^	1.56	1.7
Sunitinib	4.56^a^	4.43^a^	4.07^b^	3.06	1.4

^a^denotes those in which the *P*< 0.01

^b^denotes those in which the *P*< 0.05

#### Multidrug resistant lung cancer cell line

Compound **7e** was analyzed for cancer cell growth inhibitory activity in a sensitive NSCLC cell line (A549) and a multidrug resistant lung cancer cell line (NCI-H69AR) which expresses the ABCC1 efflux pump protein. This assessment of activity tested compounds in quadruplicate at a maximum concentration of 25 μM, followed by 10 serially diluted concentrations.

**Fig 8:**
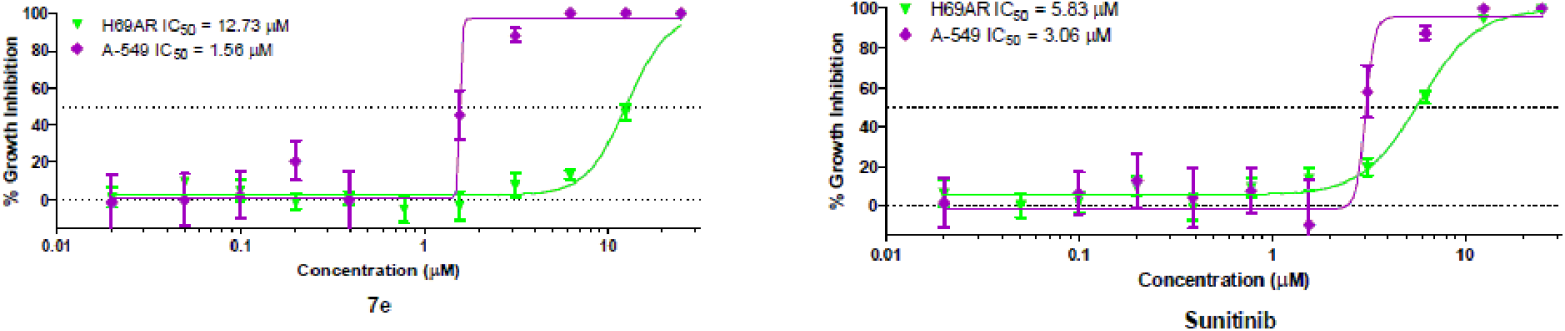
Tumor cell selectivity. Dose-dependent growth inhibition by **7e** or Sunitinib in a NSCLC cell line (A-549) compared with three non-tumorigenic cell lines: Sw-3t3 (murine fibroblasts), IEC-6 (murine inestinal epithelial cells), or MCF-10A (human mammary epithelial cells).

As indicated in Fig 8 and Table 5, compound **7e** inhibited growth in sensitive A549 and resistant NCI-H69AR cell lines with IC_50_ values of1.6 and 12.7μM, respectively. The resistant NCI-H69AR cell line was nearly eight-fold less sensitive, indicating that this compound may be subjected to efflux by ABCC1. The positive control sunitinib showed a lesser degree of resistance, i.e. the resistant NCI-H69AR cell line was 1.9-fold less sensitive.

**Table 5:** Cancer cell growth inhibitory activity of compound 7e and sunitinib toward sensitive (A-549) and resistant (NCI-H69AR) cell lines.

Compound No.	IC_50_ (μM)		Fold resistant
	Sensitive A-549	Resistant NCI-H69AR	
**7e**	1.6	12.7 ^a^	8.1
Sunitinib	3.1	5.8^a^	1.9

^a^denotes those in which the *P*< 0.01

### Evaluation of 7e-loaded microspheres

#### Encapsulation efficiency and 7e loading capacity

The prepared microspheres had high yield of 82% w/w recovery with respect to the initial amounts of polymer and **7e** used in the microsphere formulation. The average encapsulated efficiency of **7e** in PLGA microspheres was 85% ± 1.3 using a 1:10 **7e**:polymer ratio. The high encapsulation efficiency may be due to the presence of a high amount of the polymer, which can reduce **7e** loss during the fabrication process. The percentage **7e**-loading capacity was 9.84 ± 0.15.

#### Fourier transform infrared spectroscopic (FT-IR) analysis

**Fig 9:**
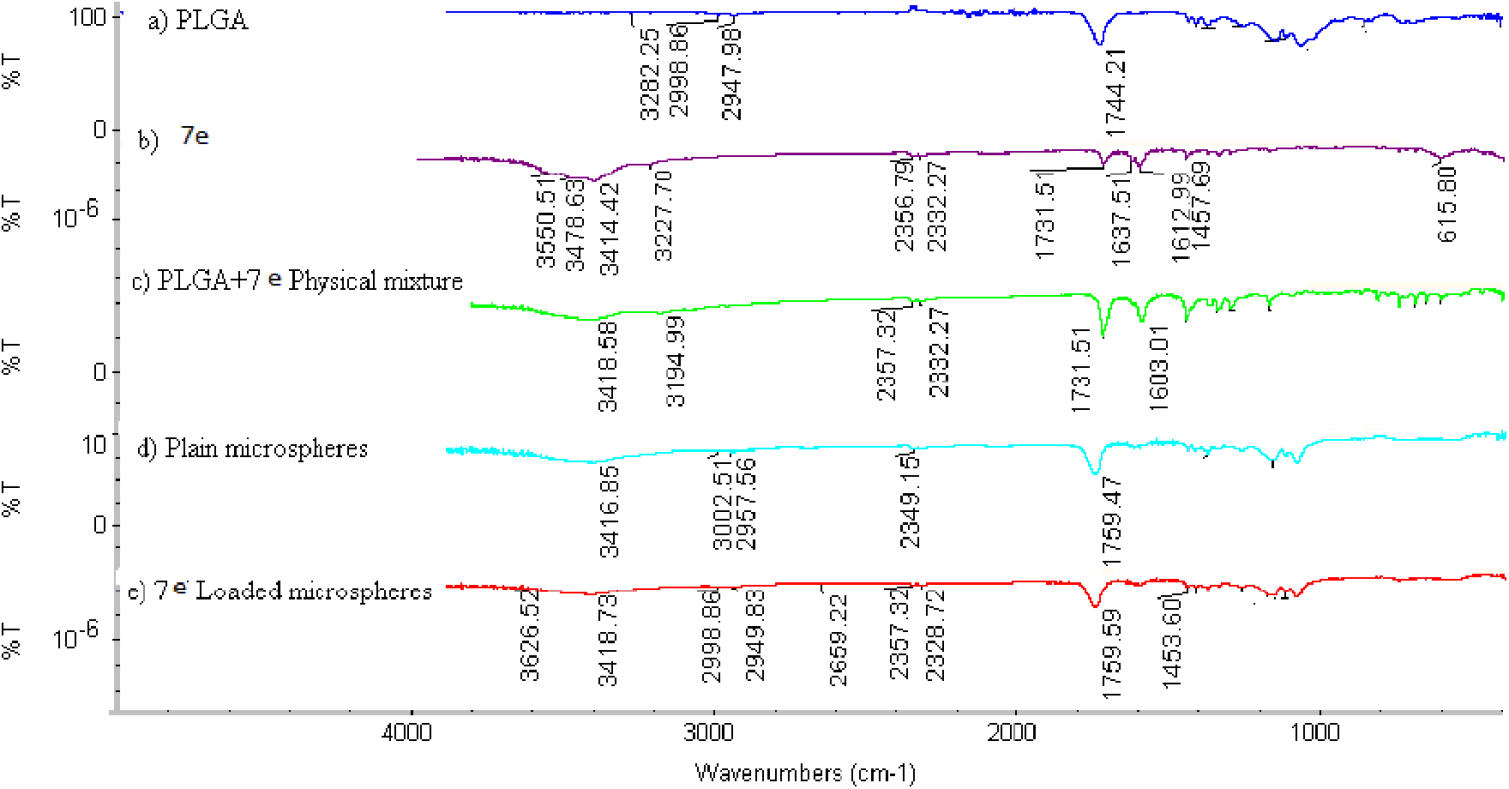
FT-IR spectrum of a) PLGA, b) Pure 7e, c) Physical mixture of the PLGA + 7e, d) Plain microspheres, and e) 7e-loaded microspheres.

The FT-IR spectra of pure **7e**, PLGA, their physical mixture, plain microspheres and **7e**-loaded microspheres were obtained to verify the chemical interaction between **7e** and the polymer. The functional group bands of **7e** remained the same in the spectra of both pure **7e** as well as in the physical mixture and the formulation. This indicated that no interaction took place between **7e** and the polymer. In the FT-IR spectrum of **7e**-loaded microspheres, it was found that there was no significant spectral shift or disappearance of the bands of **7e**in any spectrum of **7e** with the polymer, as shown in Fig 9, indicating compatibility between **7e** and the PLGA polymer.

#### Particle size determination

**Fig 10:**
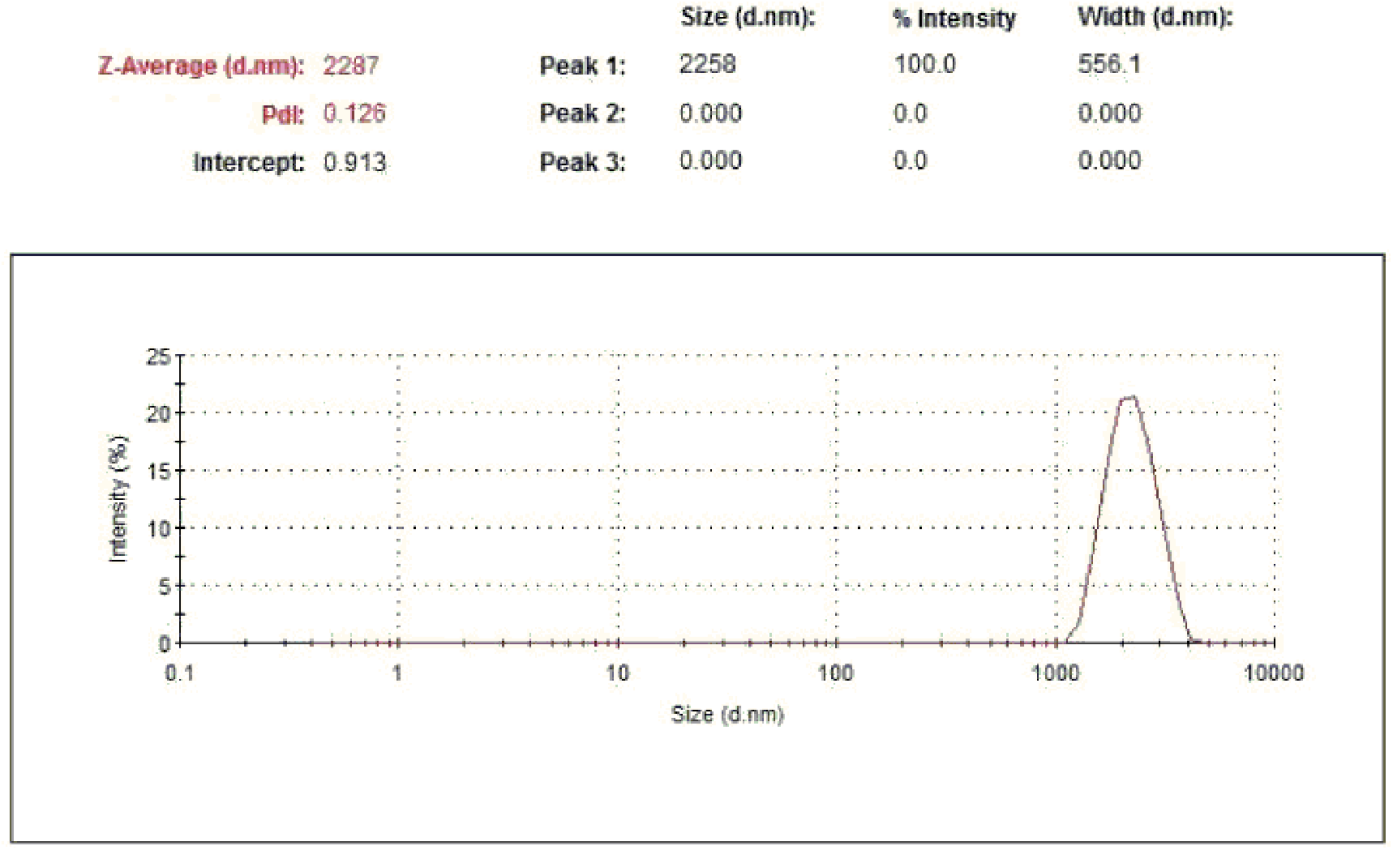
Particle size distribution of 7e-loaded PLGA microspheres.

The particle size of drug-loaded microspheres is considered an important characteristic as it could affect drug release properties. The particle size determination revealed that the produced microspheres were uniform with a size range of 1–3μm. The small polydispersity index (PdI) suggested that the size distribution of the particles was fairly monomodal (Fig 10). The loading of **7e**didnot alter the particle size, as the average particle size of the plain microspheres and the loaded microspheres was 2.26±0.2 and 2.287±0.17 μm, respectively (mean ± SD, n = 3). Fig 10 shows the particle size distribution of the **7e**-loaded microspheres around 3 μm. A narrow particle size distribution is an important aspect in passive targeting of microspheres as well as for stability issues. The results demonstrate that the particle size of the prepared microspheres was uniform.

#### Morphological analysis of 7e-loaded microspheres

**Fig 11:**
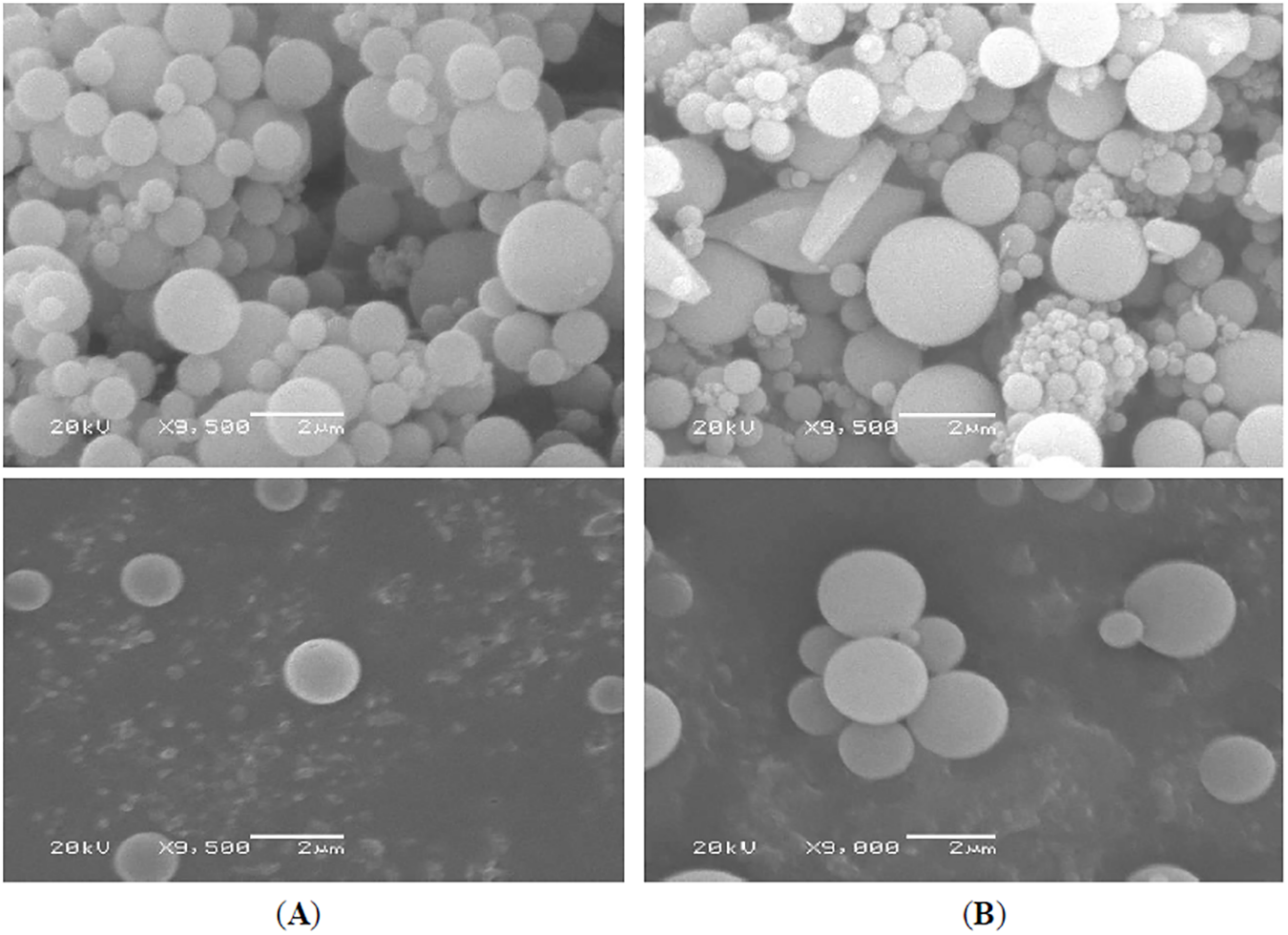
Scanning electron micrographs of (A) Free microspheres and (B) 7e-Loaded PLGA microspheres.

Scanning electron microscope (SEM) images revealed that the microspheres were intact spheres showing the formation of spherical and smooth surface microspheres with almost no pores or cavities, which may prolong the release of the encapsulated **7e** over an extended period of time. No crystals of **7e** were detected, and there was no clogging or deformation and few fragments of polymer adhering, as shown in Fig 11. No difference was observed in the morphological properties of microspheres due to the presence of compound **7e**. The particle size observed by SEM was consistent with that obtained from the Zetasizer particle size analyzer. These results indicate the efficiency of the preparation method and optimization of the preparation parameters such as the concentration and viscosity of the polymer solution. The obtained results are consistent with the previously reported studies for different drugs [40–42].

#### Drug release evaluation

Drug release from biodegradable polymeric particles occurs through a combination of several mechanisms. Generally, it occurs through desorption of surface-bound drug, diffusion of the drug through the polymeric matrix and erosion of the polymer particles.

**Fig 12:**
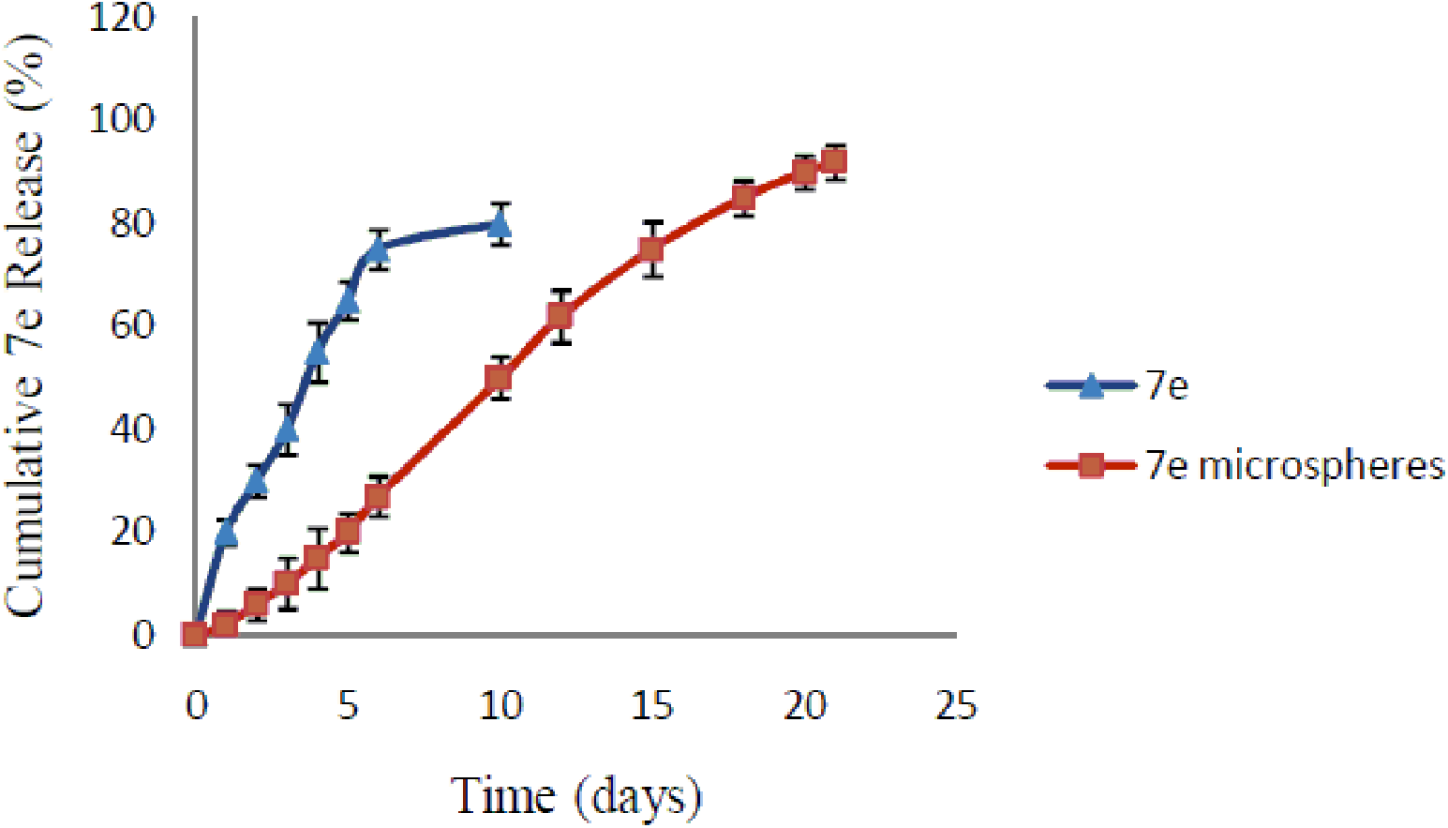
Cumulative *in vitro* release of 7e from PLGA microspheres. Each point represents the mean ± SD obtained from triplicate samples.

*In vitro* **7e** release was assessed in phosphate buffer (pH 7.4) containing a surfactant to simulate the physiological milieu and maintain sink conditions. Samples were incubated at 37°C, and the amount of **7e** was determined using a UV spectrophotometer. Fig 12 shows the release profile of raw **7e** powder and the prepared **7e**-PLGA microspheres. Linear prolonged **7e** release rates from the microspheres were observed for 21 days without an initial burst release (Fig 12). On the other hand, the release of compound **7e** from the raw powder was faster than its release from the microspheres, as 65% of **7e** was released after 5 days. This uncontrolled burst release of compound **7e** from raw powder could result in too high local concentration which could lead to severe local tissue injure and hamper appropriate delivery to the target.

It can be seen that **7e**-loaded microspheres exhibited biphasic drug release kinetics [34, 43] with an initial release up to 6% after 48 h and 27% after six days followed by cumulative release of 90% after 20 days. No initial burst release was observed, suggesting low **7e** density at the surface of the microspheres and hence the homogenous distribution of **7e** within the microspheres. The high polymer concentration led to the formation of a dense polymer matrix structure in the microspheres and no pores as observed in the SEM images, which resulted in a significant decrease in the initial burst release from the microspheres. The kinetics of **7e** release from microspheres was close to the zero order model. The correlation coefficient (r^2^) was 0.9884 and the release curve was fitted to zero order kinetics. This release pattern was due to diffusion and degradation of the microspheres.

Fig 12 shows the initial slow release of **7e** from microspheres, which could be attributed to the hydrophobicity of **7e**. Moreover, the interaction of the hydrophobic **7e** molecule with the polymer matrix *via* hydrophobic binding forces might cause a trapping of the drug inside the particles and, therefore, slower release. The rate of **7e** release from the loaded microspheres gradually increased, as 10% release was observed after three days (Fig 12).This was probably due to the diffusion of the drug, which was present at the surface of the microspheres. Thereafter, constant release was observed, which may have been due to **7e** diffusion and matrix erosion mechanisms of the biodegradable PLGA polymer. Slow drug release from microspheres maybe due high encapsulation of the drug with low swelling of polymer in the release medium, leading to slow diffusion of the drug particles from polymeric matrices. Furthermore, the degradation of PLGA 50:50 is slow. Therefore, the release of **7e** from microspheres may depend on drug diffusion, the PLGA surface and bulk erosion or swelling [34].Conclusively, compound **7e** was slowly and continuously released from the PLGA microspheres with no obvious burst release. This behavior is consistent with a previous study using PLGA microspheres for sustained drug release [44] and with several anticancer drugs incorporated into microspheres [33, 40, 45–47]. This formula was used for further studies on the human lung cancer cell line A549.

#### Cell viability assay

The human lung cancer A549 cell line was incubated with various concentrations (0.8, 1.6, 3.13, 6.25, 12.5, 25 and 50 μM) of both free **7e** and **7e**-loaded microspheres to evaluate the anti-proliferative activity by assessing their effect on cell viability. Unloaded PLGA microspheres (lacking compound **7e**) were coincubated with the same cell line to show that tumor growth was not inhibited due to the PLGA microspheres alone.

**Fig 13:**
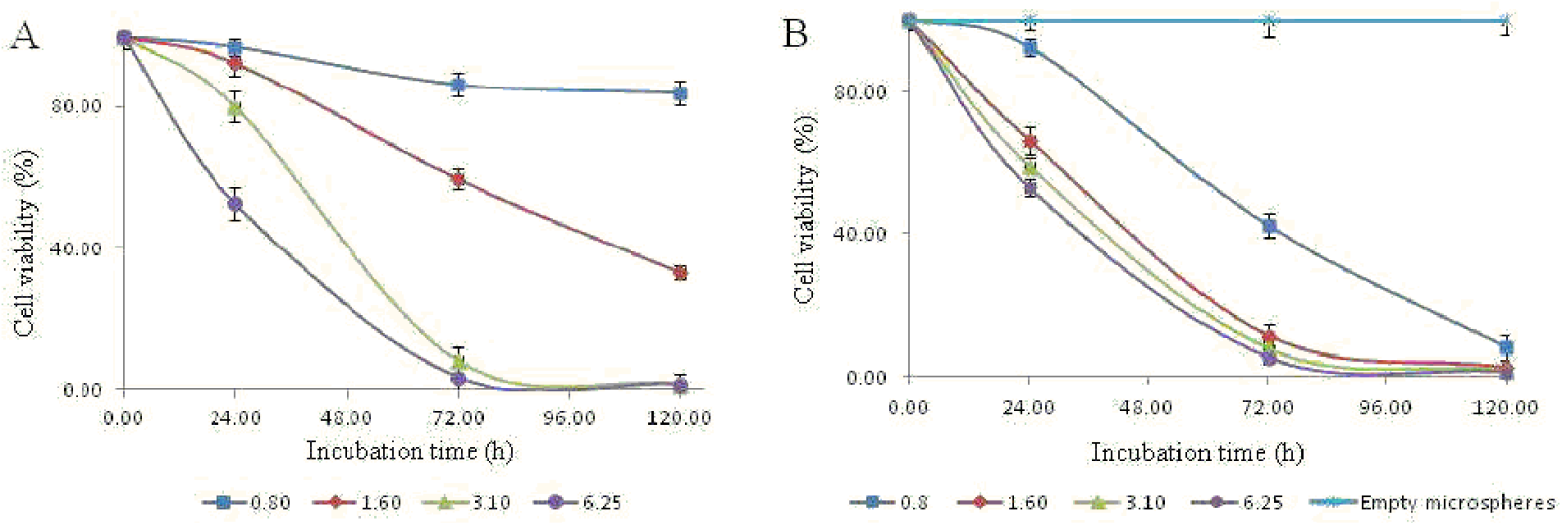
Cell viability of A549 cells incubated with free 7e (A) and 7e-loaded microspheres (B) at 0.8, 1.6, 3.13, and 6.25 μM drug concentrations after 24, 72, and 120 h incubation time.

In Fig 13A, the cytotoxicity of free **7e** was evaluated at0.8, 1.6, 3.13 and 6.25 μM. After 24 h of incubation time, no cytotoxicity was observed at low concentrations (0.8 and 1.6 μM), while after 72 h, only a 30% reduction in cell viability could be seen at 1.6 μM. Moreover, at 3.1 μM, 20 and 70% reductions in the cell viability were noted after 24 h and 72 h of incubation, respectively, and almost total inhibition was observed after 120 h of incubation. A remarkable reduction in cell viability was observed at 6.25 μM = after 24 h of incubation and was maintained for 120 h of incubation. Therefore, the cytotoxicity effect of free **7e** needs higher concentrations to obtain minimal to no growth of the human lung cancer cell lineA549.

Fig 13B illustrates the obtained results when **7e**-loaded microspheres were incubated with the human lung cancer cell lineA549for incubation periods and concentrations similar to that of the free **7e**. There was no cytotoxicity observed at 0.8 μM after 24 h of incubation, but at 1.6, 3.13 and 6.25 μM, 30, 40 and 50% reductions in cell viability were observed, respectively. After 72 h of incubation, cell viability was decreased to 10, 7 and 5%, respectively, for the same concentrations. This assay demonstrated that entrapped **7e** was more effective in arresting cell growth as compared with free **7e**. The enhanced cytotoxicity of **7e**-loaded microspheres was observed with increasing incubation time in a concentration dependent manner. Therefore, the decrease in percentage cell viability was not immediate but rather gradual and continuous due to the polymer that allowed for slow release of **7e** from microspheres and hence its gradual cytotoxic effect. The extended activity of **7e** from **7e**-loaded microspheres might be explained by the fact that they can be adsorbed onto the cell membrane, generating a drug concentration gradient near the cell surface that could favor **7e** to penetrate into the cell. Furthermore, the cell can phagocytize **7e**-loaded microspheres, allowing **7e** to be released inside the cytoplasm, thus contributing to a sustained **7e** concentration [48].

**Fig 14:**
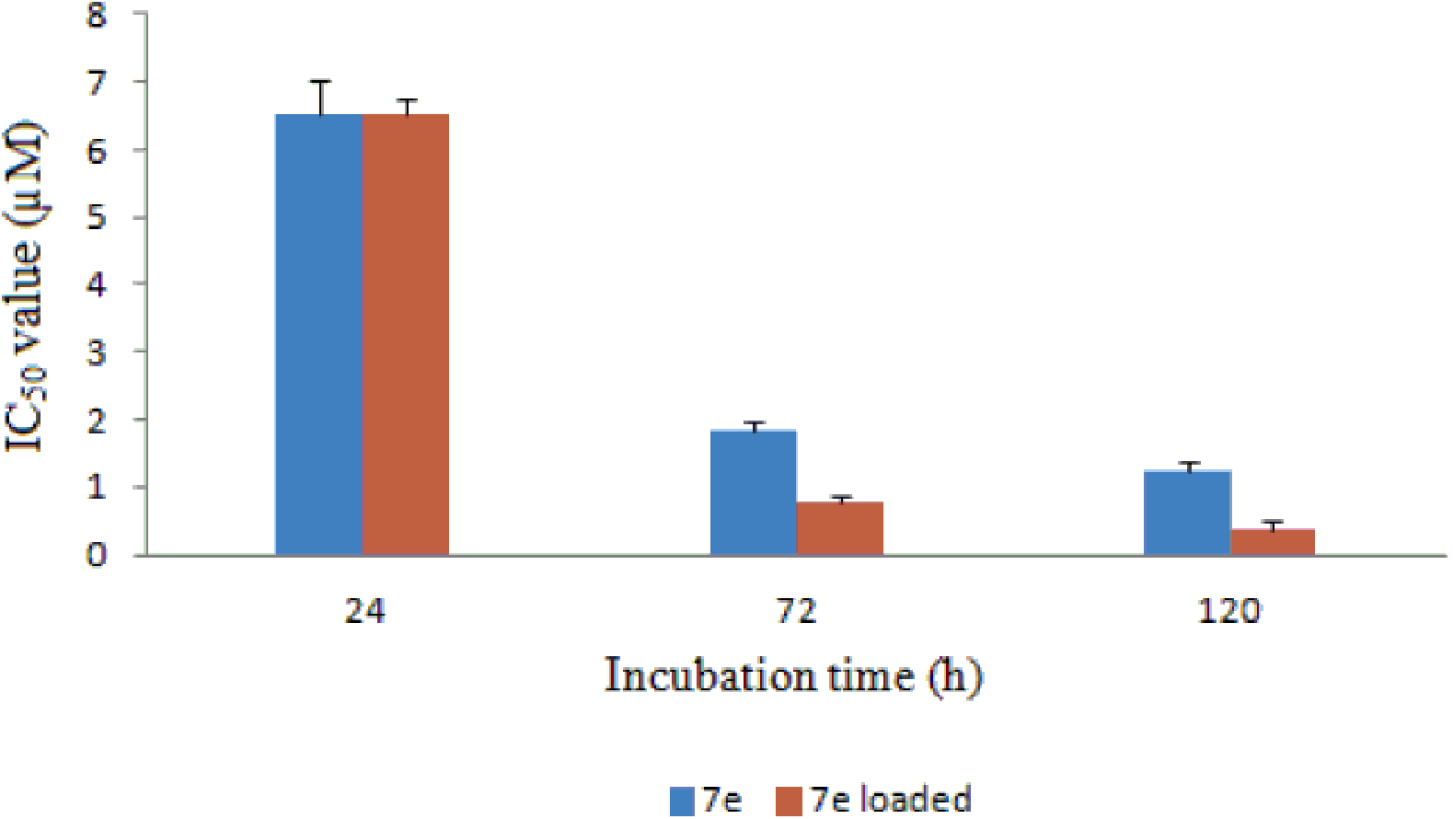
The IC_50_ values of the free 7e and 7e-PLGA loaded microspheres.

Quantitative *in vitro* cytotoxicity of **7e**-loaded microspheres was investigated *via* the determination of theIC_50_ value toward A549cells as compared with that of free **7e.** The IC_50_ value was almost the same for both the free **7e** (IC_50_ value = 6.51 μM) and the **7e**-loaded microspheres (IC_50_ value = 6.50 μM) after 24 h of incubation (Fig 14). Increasing the incubation time of **7e**-loaded microspheres to 72 and 120 h resulted in a significant reduction in its IC_50_ value by approximately 85 and 90%, respectively. On the other hand, the IC_50_ value of **7e**-loaded microspheres was reduced to approximately 60 and 70% after 72 and 120 h, respectively, as compared with the IC_50_ values of free **7e**. Statistical analysis revealed that these values were significantly different (*P*< 0.05) from the IC_50_ values of free **7e**.

The enhanced**7e** activity mediated by its incorporation into microspheres can be attributed to its sustained release, as shown in Fig 12. Microspheres can act as a reservoir for **7e**, protecting the drug from hydrolysis and provide not only sustained release of the drug, but also contribute to the maintenance of drug activity. Several reported studies have emphasized that incorporation of anticancer drugs in such delivery systems improves their cytotoxic effects[49, 50].

## Conclusions

The synthesis and molecular characterization using different spectroscopic tools of certain isatin-based hybrids **4a-o** and **7a-e** are described. Compounds **4a-o** and **7a-e** were subjected to *in vitro* anti-proliferative assessment against HT-29 (colon), ZR-75 (breast) and A549 (lung) human cancer cell lines. The most active congeners in the preliminary *in vitro* anti-proliferative screening, compounds **7b**, **7d** and **7e**, showed average IC_50_ values of 4.77, 3.39 and 2.37 μM, respectively, as compared with the reference drug sunitinib which exhibited an average IC_50_ value of 8.11 μM towards the tested human cancer cell lines. Compound **7e** was selected for deeper pharmacological testing to gain insight into its possible *in vitro* anti-proliferative mechanism of action. It increased caspase activity by 2.4- and 1.85-fold between 4 and 8 h of treatment, respectively, at 10 μM. Moreover, compound **7e** decreased the percentage of cells in the G1 phase of the cell cycle with a corresponding increase in the S-phase. The increase in S-phase cells without a concomitant G2/M phase fraction may indicate a very rapid onset of apoptosis. In addition, compound **7e**increased p-Tyr levels by nearly two-fold with an apparent IC_50_ value of 3.8 μM, which is consistent with its growth inhibitory IC_50_ value. Compound **7e** was incorporated into biodegradable PLGA microspheres to improve its *in vitro* anti-proliferative activity. The results of the *in vitro* anti-proliferative assay indicated that **7e**-loaded PLGA microspheres exhibited better cytotoxicity as compared with free **7e** toward the human lung cancer cell line A549 after 3 and 5 days of incubation.

## Supporting information

S1 File Experimental protocols of the pharmacological evaluation of the synthesized target compounds. (DOCX)

S2 File Representative examples of the NMR (^1^H and ^13^C) spectra of the synthesized target compounds. (DOC)

S3 File Synthetic pathway to achieve the target compounds 4a-o. (DOCX)

S4 File Synthesis of the target compounds 7a-e. (DOCX)
