# Supplementary material for: New hydrazonoindolin-2-ones: Synthesis, exploration of the possible anti-proliferative mechanism of action and encapsulation into PLGA microspheres
Source: PLoS One. 2017 Jul 25;12(7):e0181241. doi: 10.1371/journal.pone.0181241 (PMC5526551; doi:10.1371/journal.pone.0181241)
Supplement: S1 File — (DOCX) [file pone.0181241.s001.docx]

**New hydrazonoindolin-2-ones**: **synthesis, exploration of the possible anti-proliferative mechanism of action and encapsulation into PLGA microspheres**

Mohamed I. Attia^1,2^, Wagdy M. Eldehna^3^, Samar A. Afifi^4,5^, Adam B. Keeton^6^, Gary A. Piazza^6^ and Hatem A. Abdel-Aziz^7^

^1^Department of Pharmaceutical Chemistry, College of Pharmacy, King Saud University, P.O. Box 2457, Riyadh 11451, Saudi Arabia

^2^Medicinal and Pharmaceutical Chemistry Department, Pharmaceutical and Drug Industries Research Division, National Research Centre (ID: 60014618), El Bohooth Street, Dokki, Giza 12622, Egypt

^3^Department of Pharmaceutical Chemistry, Faculty of Pharmacy, Kafrelsheikh University, 33516 Kafrelsheikh, Egypt

^4^Department of Pharmaceutics, National Organization for Drug Control and Research, P.O. Box 35521, Giza 12561, Egypt

^5^Department of Pharmaceutics, College of Pharmacy, King Saud University, P.O. Box 2457, Riyadh 11451, Saudi Arabia

^6^Department of Oncologic Sciences and Pharmacology, Drug Discovery Research Center, Mitchell Cancer Institute, University of South Alabama,1660 Springhill Avenue, Mobile, AL 36604-1405, USA

^7^Department of Applied Organic Chemistry, National Research Centre, (ID: 60014618), El Bohooth Street, Dokki, Giza 12622, Egypt

**Pharmacological investigations**

**Anti-proliferative activity**

A549, HT-29, ZR-75 were obtained from the American Type Culture Collection (ATCC). Cells were harvested from 90-95% confluent 10 cm dishes, and diluted in growth media (RPMI-1640 supplemented with 5% fetal bovine serum). An appropriate concentration of cells were prepared to add 1,250 cells in a volume of 25 µL per well in tissue culture treated Griener 384-well microplates, then incubated overnight at 37°C. Compound **7e** was diluted to a concentration of 30 µM in DMSO. Concentrated dosing solutions of twice the final concentration were prepared by dilution of the DMSO stocks 1:500 in growth media. Two-fold serial dilutions were then prepared in medium containing 0.2% DMSO to maintain a constant concentration of vehicle throughout the concentration range of the compound. Each of these was added in an equal volume (25 µL) to four wells on each cell assay plate, along with the vehicle control. The highest concentration for serial dilutions of the positive control (sunitinib) was prepared exactly as the experimental compound **7e**. Cells were incubated with the compounds for a further 72 h.

At the end of the treatment period, assay plates were allowed to cool to RT for 10 min prior to the addition of 25 µL per well Promega CellTiterGlo reagent, followed by additional 10 min incubation at RT. The resulting luminescence was quantitated using a Molecular Devices Spectramax Paradigm.

Percent growth inhibition was calculated as follows, where 100 represents the viability of vehicle treated control samples:

100 - (100 x (sample) ) (1)

(vehicle control)

Thus, on average, growth inhibition of the vehicle control samples equals zero.

The potency of compounds was determined using the non-linear dose response algorithm (four-parameter logistic fit) with GraphPad Prism 5 software.

**Apoptosis and caspase 3/7 activity**

A549 cells were harvested from a 90% confluent 10 cm dish and diluted in growth medium. An appropriate concentration of cells were prepared to add 1,250 cells in a volume of 25 µL per well in tissue culture treated Corning 384-well microplates, then incubated overnight at 37°C. A single cluster of four wells containing no cells was included to serve as a background (no cell) control. Compound **7e** was diluted in DMSO to concentrations 3000-fold above the IC_50_ value.

Concentrated dosing solutions of twice the final concentration were prepared by dilution of the DMSO stocks 1:500 in growth media. Three-fold dilutions of each dosing medium was then prepared in medium containing 0.2% DMSO to maintain a constant concentration of vehicle throughout the concentration range of the compound. Each of these was added in equal volume (25 µL) to four wells on each cell assay plate, along with the vehicle control. Cells were incubated with the compounds for a further 48 h. After 24 h, a separate group of cells was treated identically for 24 h. Likewise, groups of cells were treated for durations of 16, 8, 4 and 2 h.

At the end of the treatment period, assay plates were allowed to cool to RT for 10 min prior to addition of 25 µL per well Promega CaspaseGlo 3/7 reagent, followed by an additional 10 min incubation at RT. The resulting luminescence was quantitated using a Molecular Devices Spectramax Paradigm.

Apoptosis activity as a function of caspase 3/7 was calculated as follows:

The average mean value of the background “no cell” control sample wells were subtracted from all vehicle and test compound wells.Fold-change in caspase activity was then calculated by dividing the mean sample value for each treatment time point by the mean vehicle value for the corresponding time.

Fold Change = ____(sample)­­­­___ (2)

(vehicle control)

Thus, on average, caspase 3/7 activity of vehicle control samples equals one.

Fold-change induced by the two tested concentrations for each compound were plotted over a time course with GraphPad Prism 5 software.

**Cell cycle effects**

A549 cells were harvested from a 90% confluent 10 cm dish and diluted in growth medium. An appropriate concentration of cells were prepared to add 5,000 cells in a volume of 100 µL per well in tissue culture treated PerkinElmer optical bottom ViewPlate microplates, then incubated overnight at 37°C. The following day, compound **7e** and the positive control, sunitinib, were diluted in DMSO to concentrations 1000-fold above the maximum tested concentration.

Concentrated dosing solutions of twice the final concentration were prepared by dilution of the DMSO stocks 1:500 in growth media. Three-fold dilutions of each dosing medium were then prepared in medium containing 0.2% DMSO to maintain a constant concentration of vehicle throughout the concentration range of each compound. Each of these was added in equal volume (100 µL) to each well on the assay plate, along with the vehicle control. One plate of cells was incubated with the compounds for a further 24 h, and a separate plate was incubated with the compounds for 48 h.

At the end of the respective treatment periods, 150 µL per well of neutral buffered formalin fixative (10% paraformaldehyde) was rapidly added to each plate and incubated at room temperature for 20 min. The fixative was removed by washing thrice with phosphate buffered saline (PBS). Cell membranes were permeabilized and non-specific binding sites were blocked by incubation with 0.3% Triton X-100 and 5% FBS in PBS for 1 h. Phosphorylated Rb protein (p-Rb) was detected by 1 h incubation with anti-p-Rb rabbit monoclonal antibody (Cell Signaling #8516) diluted 1:1600 in antibody dilution buffer (1% bovine serum albumin and 0.3% Triton X-100 in PBS). Unbound antibody was removed by washing thrice with PBS. Anti-Phospho Ser807/811-Rb antibody (Cell Signalling cat #8516) was detected by 1 h incubation with goat anti-rabbit AlexaFluor 488 conjugate (InVitrogen cat #A-11008) diluted 1:500 in antibody dilution buffer. Unbound antibody was removed by washing thrice with PBS. Nine fields per well were immediately imaged in a Molecular Devices ImageXpress MicroXL automated fluorescent microscope system using a 10x magnification objective lens. DAPI and FITC filter cubes, respectively, were used to visualize nuclei and immunolabeled P-Rb residues. Identical autofocus and exposure parameters were used for every sample. Fluorescent micrographs were analyzed using Molecular Devices MetaXpress multi-wavelength scoring image analysis program to quantitate Rb phosphorylation. The mean nuclear average intensity of P-Rb staining (RFU) per cell was reported and has been graphed using GraphPad Prism statistical and graphing software. Where appropriate, an IC_50_ value has been reported to reflect potency of the compound effect on P-Rb.

DAPI (1 µg/mL) fluorescent dye was incubated with fixed cells to quantitate double stranded DNA within each cell nucleus to determine cell cycle distribution. DNA content in each cell was determined by total DAPI fluorescent intensity by high throughput image acquisition and analysis. Fluorescent intensities were plotted on histograms with threshold intensity values assigned to each cell cycle classification using the MetaXpress cell cycle analysis program. Results from each treatment were reported as average cell number in each of nine fields, as well as the percentage of cells within each phase of the cell cycle. Each of these parameters was analyzed using GraphPad Prism, and, where appropriate, IC_50_ values have been reported.

**Selectivity**

A549, IEC-6, MCF-10A and 3t3 cells were harvested from 90-95% confluent cultures in 10 cm dishes, and diluted in growth media recommended by the suppliers as follows: A549 cells were cultured in RPMI-1640 medium supplemented with 5% fetal bovine serum (FBS). IEC-6 cells were obtained from ATCC and cultured in Dulbecco’s modified Eagle’s medium (DMEM) supplemented with 10% FBS and 0.1 unit/mL recombinant insulin. Swiss 3t3 cells were obtained from ATCC and cultured in DMEM supplemented with 10% FBS. MCF-10Acells were obtained from ATCC and cultured in a mixture DMEM and F12 media supplemented with 5% fetal bovine serum (FBS), 20 ng/mL recombinant epidermal growth factor, 10 µg/mL insulin, 0.5 µg/mL hydrocortisone and 100 ng/mL cholera toxin. An appropriate concentration of cells was prepared to add 1,250 cells in a volume of 25 µL per well in tissue culture treated BD 384-well microplates, then incubated overnight at 37 °C.

Compound **7e** and the positive control, sunitinib, were diluted to a concentration of 25 µM in DMSO. Concentrated dosing solutions (double the final concentration) were prepared by dilution of the DMSO stocks 1:500 in growth medium. Two-fold serial dilutions were then prepared in medium containing 0.2% DMSO to maintain a constant concentration of vehicle throughout the concentration range of the compound. Each of these was added in equal volume (25 µL) to four wells on each cell assay plate, along with vehicle control. The highest concentration for serial dilutions of the positive control sunitinib was prepared exactly as the experimental compounds. Cells were incubated at 37 °C with the compounds for a further 72 h.

At the end of the treatment period, assay plates were allowed to cool to RT for 10 min prior to addition of 25 µL per well Promega CellTiterGlo reagent, followed by an additional 10 min incubation at RT. The resulting luminescence was quantitated using a Molecular Devices Spectramax Paradigm.Percent growth inhibition was calculated using equation (1). The potency of compounds was determined using the non-linear dose response algorithm (four-parameter logistic fit) with GraphPad Prism 5 software.

**Multidrug resistant lung cancer cell line**

A549 and H69AR cells were harvested from 90-95% confluent 10 cm dishes, and diluted in growth media. An appropriate concentration of cells was prepared to add 1,250 cells in a volume of 25 µL per well in tissue culture treated BD 384-well microplates, then incubated overnight at 37 °C.

Compound **7e** was diluted to a concentration of 25 µM in DMSO. Concentrated dosing solutions (double the final concentration) were prepared by dilution of the DMSO stocks 1:500 in growth medium. Two-fold serial dilutions were then prepared in medium containing 0.2% DMSO to maintain a constant concentration of vehicle throughout the concentration range of the compound. Each of these was added in equal volume (25 µL), to four wells on each cell assay plate, along with the vehicle control. The highest concentration for serial dilution of the positive control compound sunitinib was prepared exactly as the experimental compound **7e**. Cells were incubated at 37°C with the compounds for a further 72 h. At the end of the treatment period, assay plates were allowed to cool to RT for 10 min prior to addition of 25 µL per well Promega CellTiterGlo reagent, followed by an additional 10 min incubation at RT. The resulting luminescence was quantitated using a Molecular Devices Spectramax Paradigm.

Percent growth inhibition was calculated using equation (1). The potency of compounds was determined using the non-linear dose response algorithm (four-parameter logistic fit) with GraphPad Prism 5 software.
