# Supplementary material for: New hydrazonoindolin-2-ones: Synthesis, exploration of the possible anti-proliferative mechanism of action and encapsulation into PLGA microspheres
Source: PLoS One. 2017 Jul 25;12(7):e0181241. doi: 10.1371/journal.pone.0181241 (PMC5526551; doi:10.1371/journal.pone.0181241)

**New hydrazonoindolin-2-ones**: **synthesis, exploration of the possible anti-proliferative mechanism of action and encapsulation into PLGA microspheres**

Mohamed I. Attia^1,2^, Wagdy M. Eldehna^3^, Samar A. Afifi^4,5^, Adam B. Keeton^6^, Gary A. Piazza^6^ and Hatem A. Abdel-Aziz^7^

^1^Department of Pharmaceutical Chemistry, College of Pharmacy, King Saud University, P.O. Box 2457, Riyadh 11451, Saudi Arabia

^2^Medicinal and Pharmaceutical Chemistry Department, Pharmaceutical and Drug Industries Research Division, National Research Centre (ID: 60014618), El Bohooth Street, Dokki, Giza 12622, Egypt

^3^Department of Pharmaceutical Chemistry, Faculty of Pharmacy, Kafrelsheikh University, 33516 Kafrelsheikh, Egypt

^4^Department of Pharmaceutics, National Organization for Drug Control and Research, P.O. Box 35521, Giza 12561, Egypt

^5^Department of Pharmaceutics, College of Pharmacy, King Saud University, P.O. Box 2457, Riyadh 11451, Saudi Arabia

^6^Department of Oncologic Sciences and Pharmacology, Drug Discovery Research Center, Mitchell Cancer Institute, University of South Alabama,1660 Springhill Avenue, Mobile, AL 36604-1405, USA

^7^Department of Applied Organic Chemistry, National Research Centre, (ID: 60014618), El Bohooth Street, Dokki, Giza 12622, Egypt

**Representative examples of the NMR (^1^H and ^13^C) spectra of the synthesized compounds.**


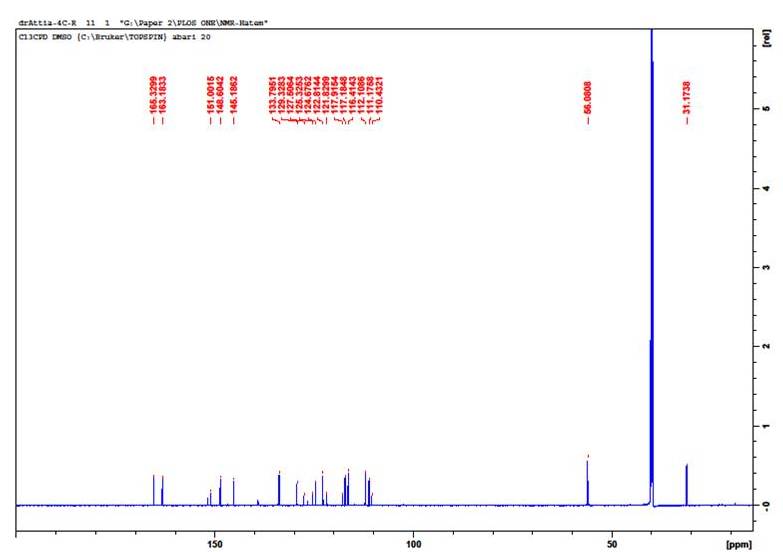


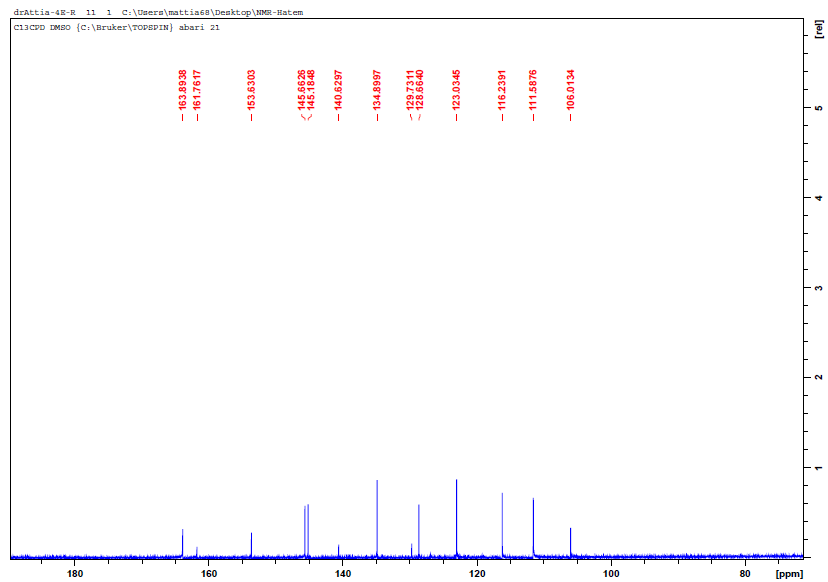


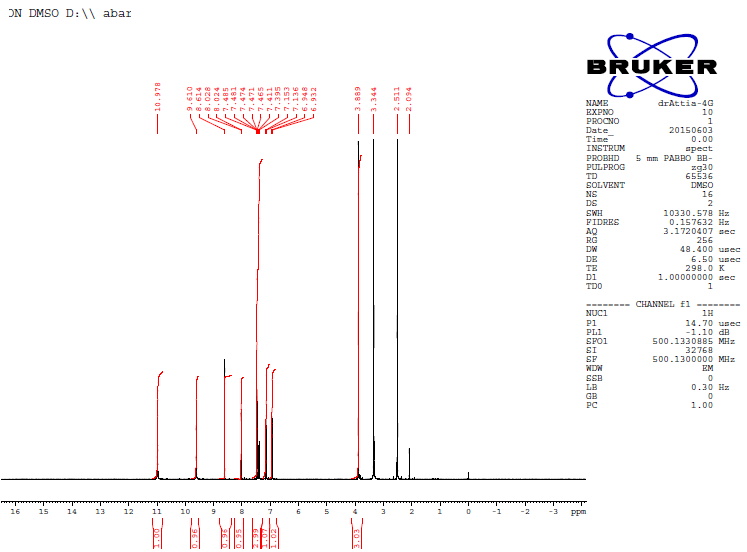


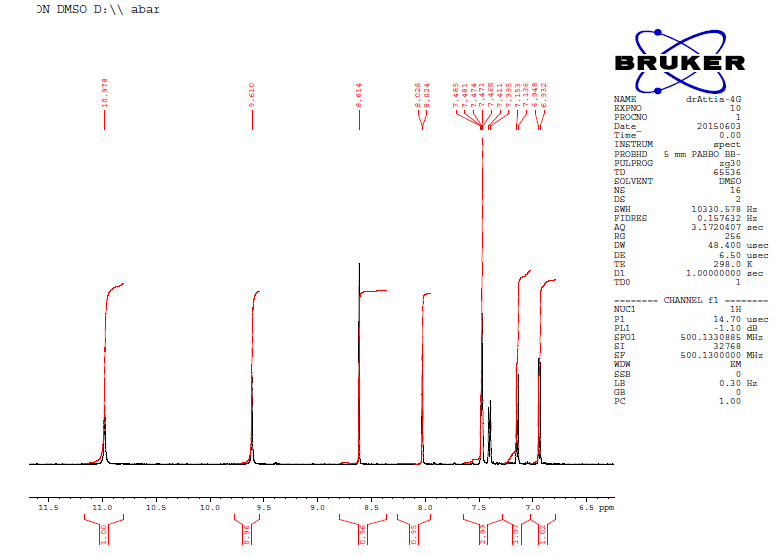


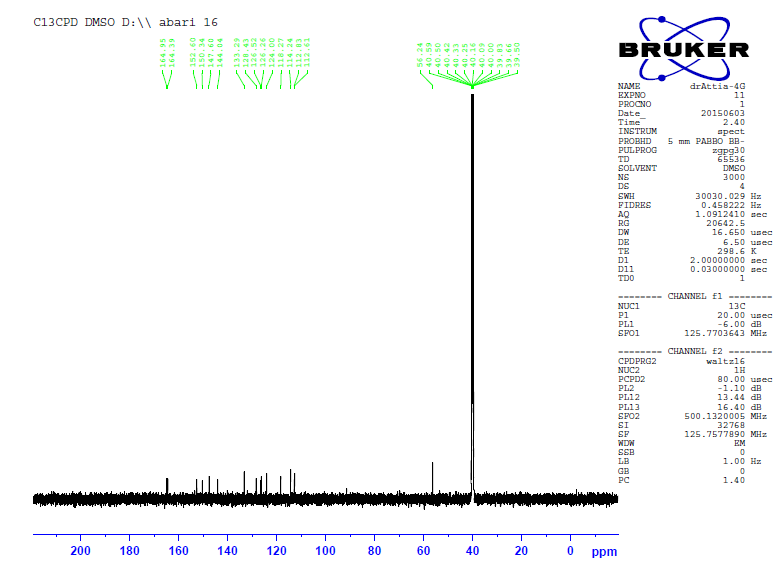


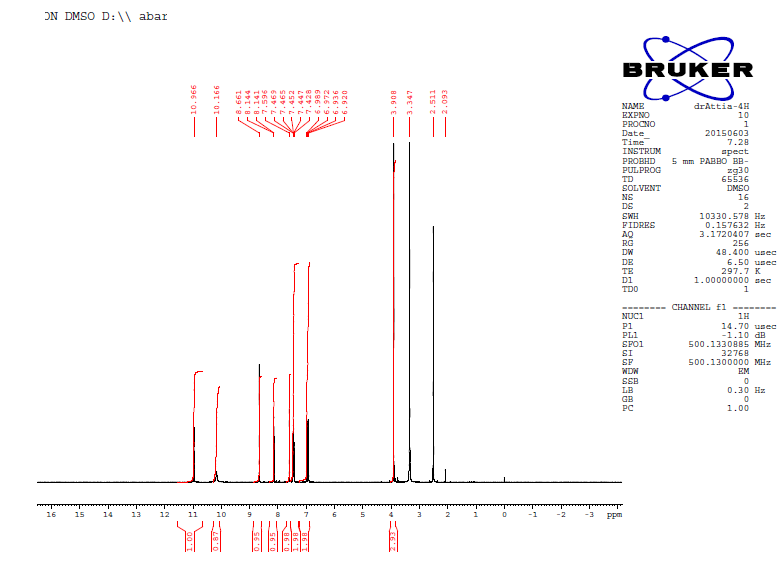


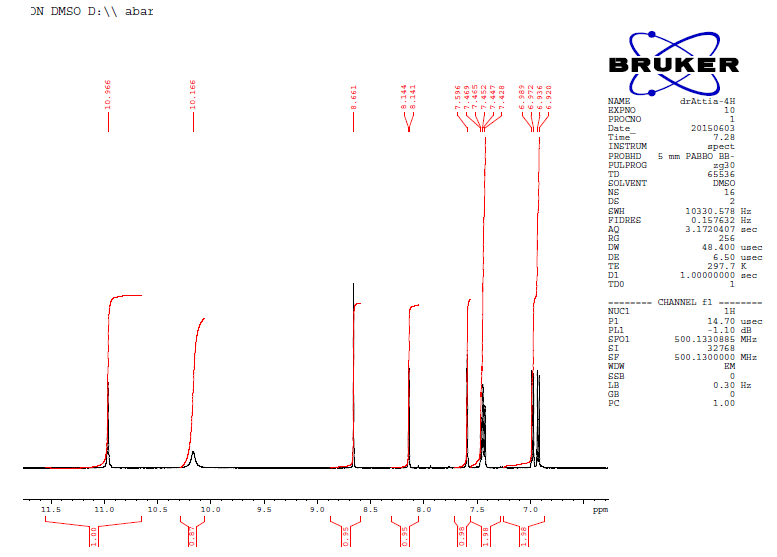


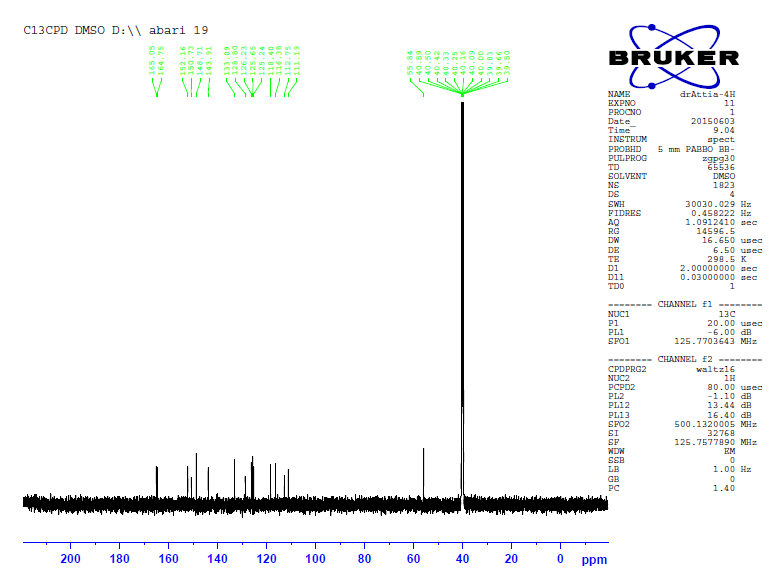


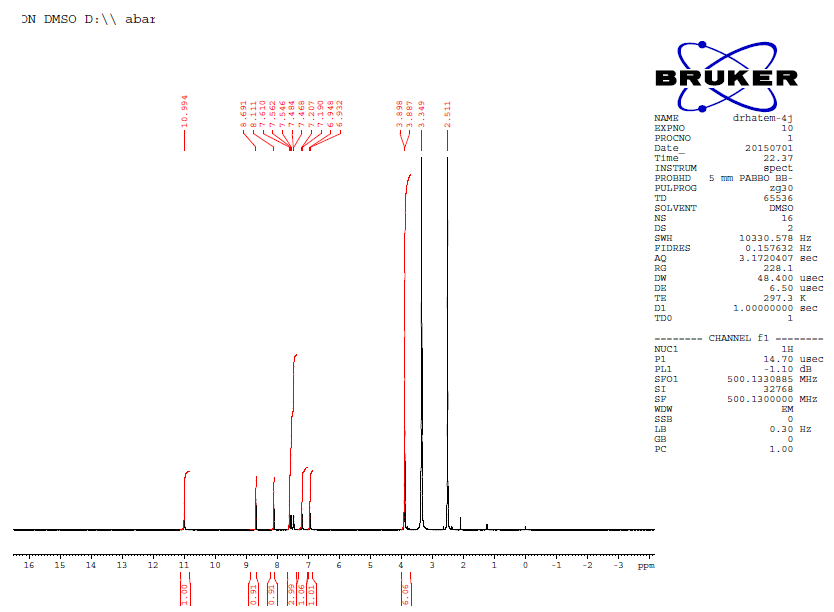


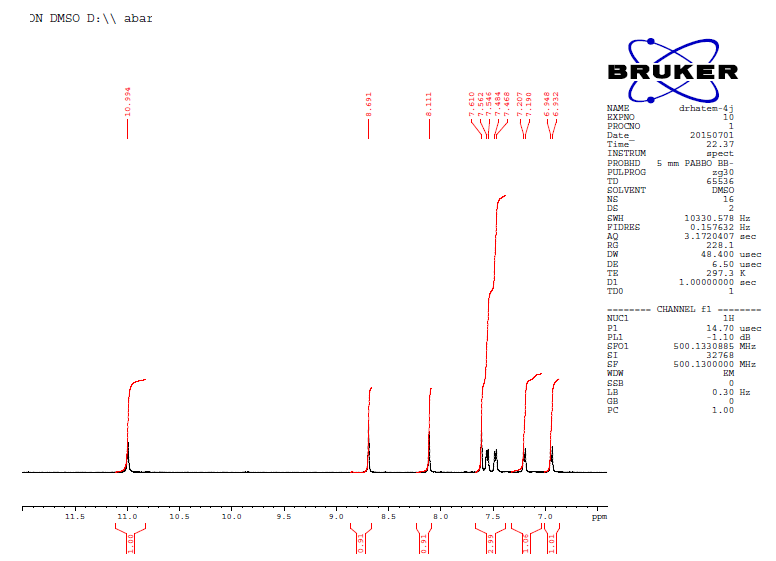


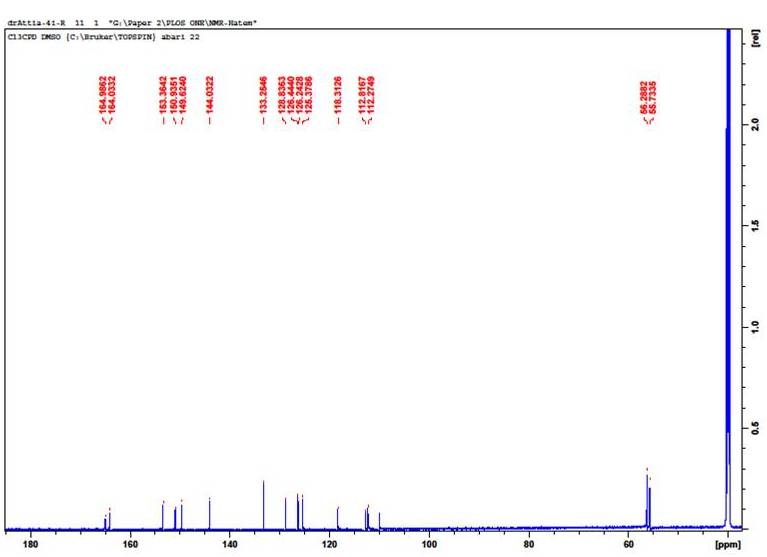


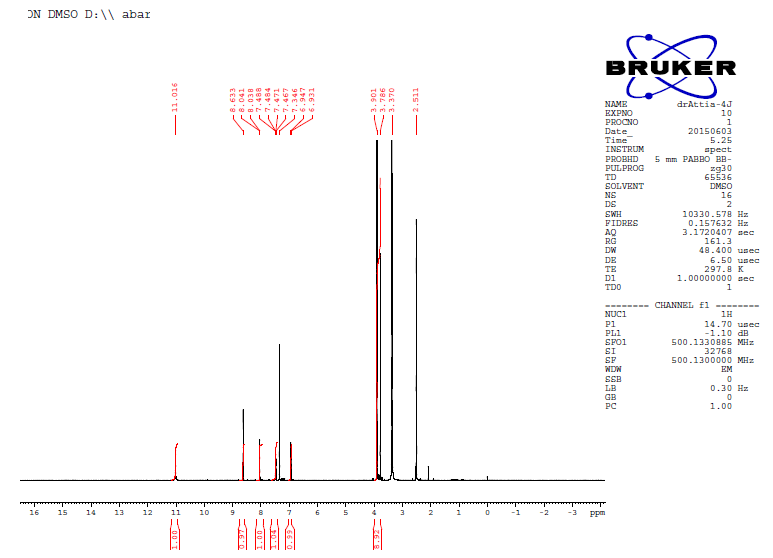


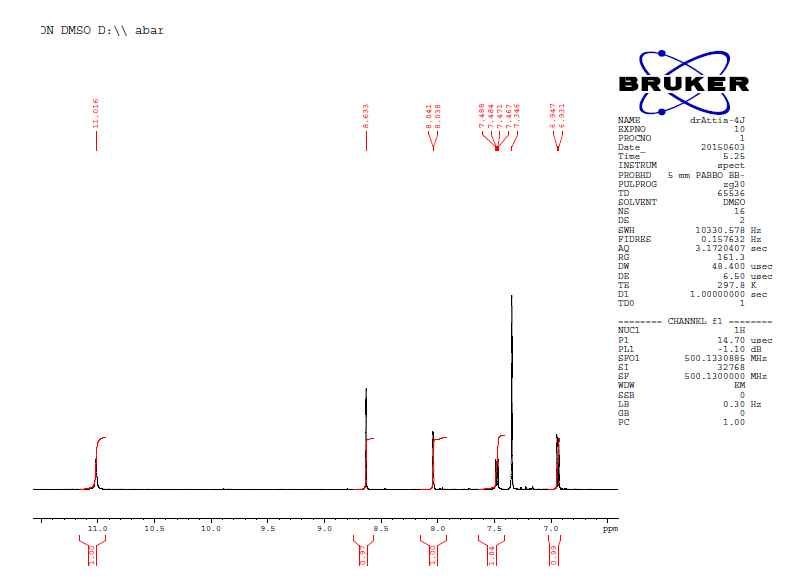


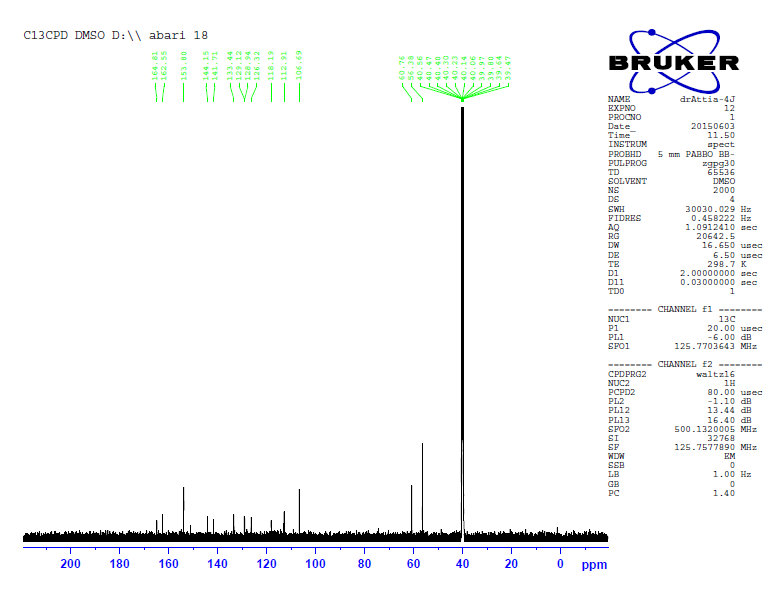


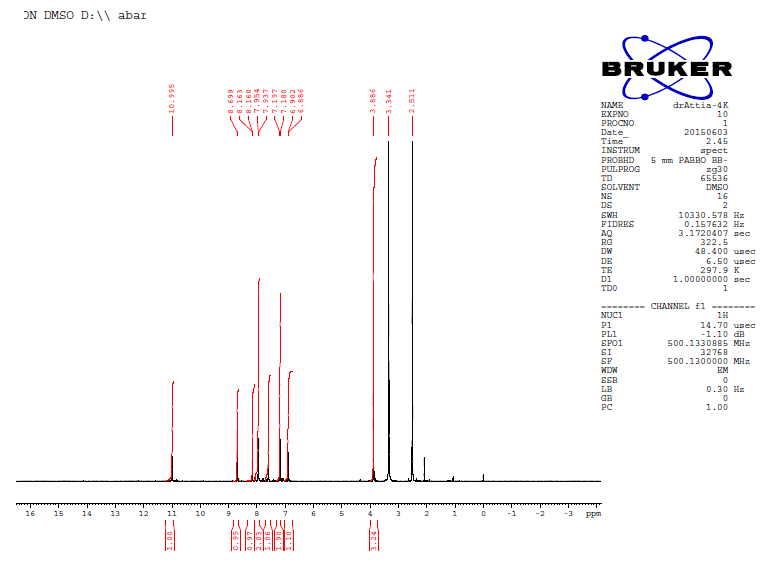


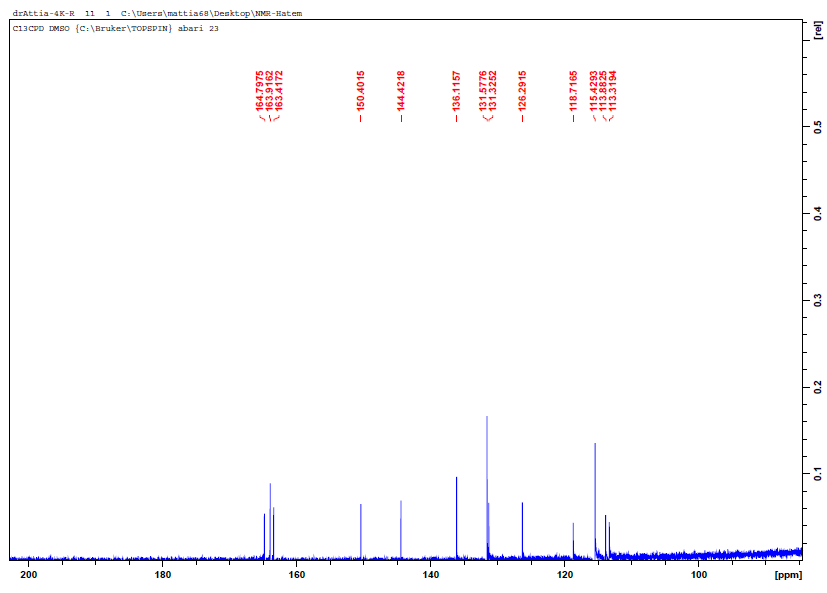


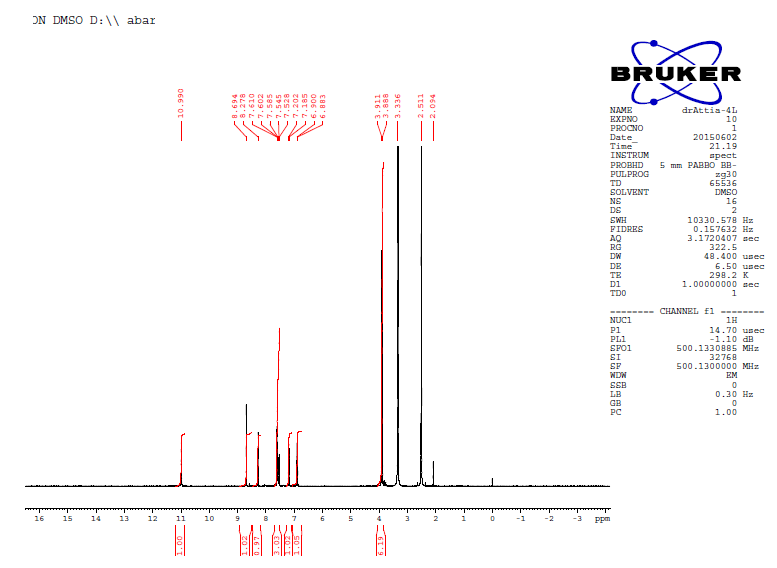


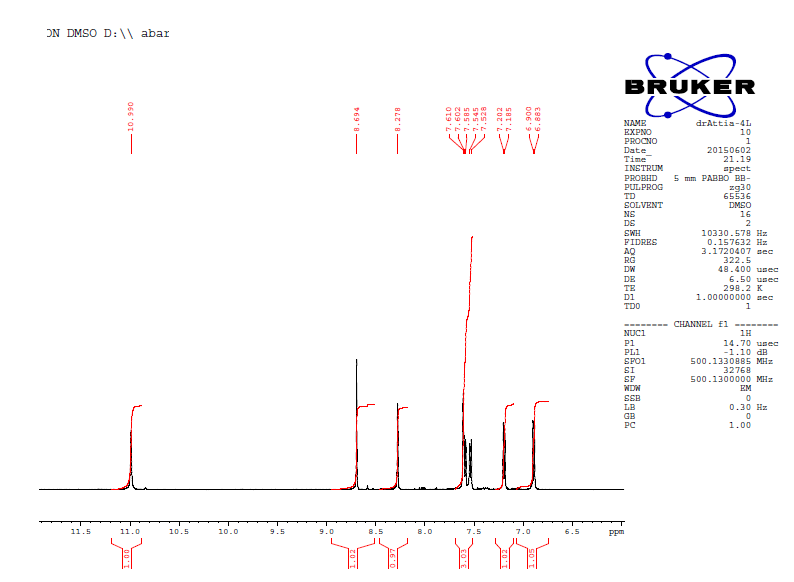


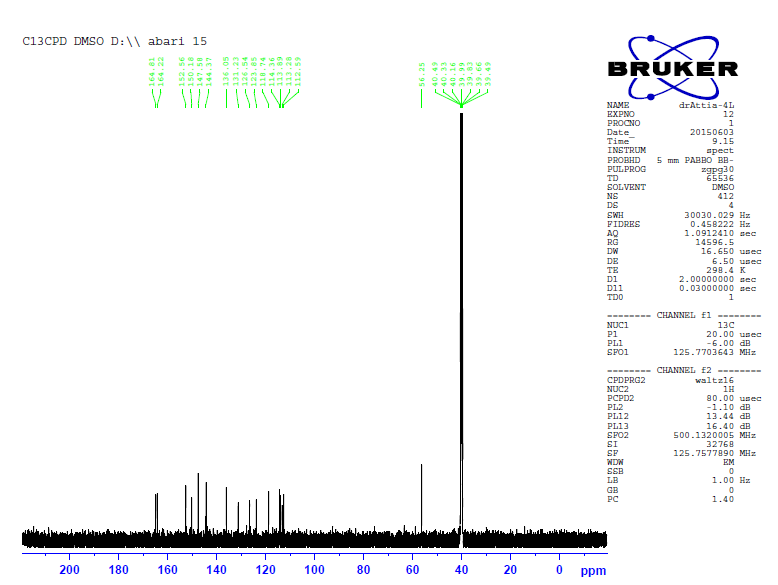


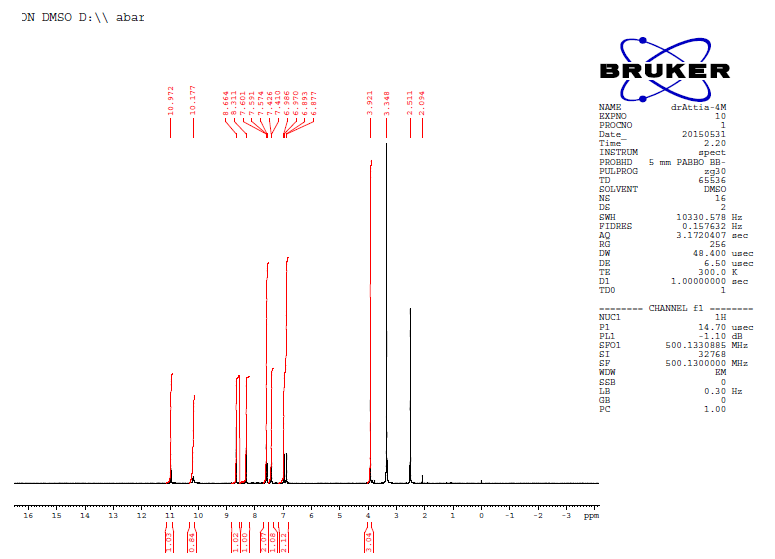


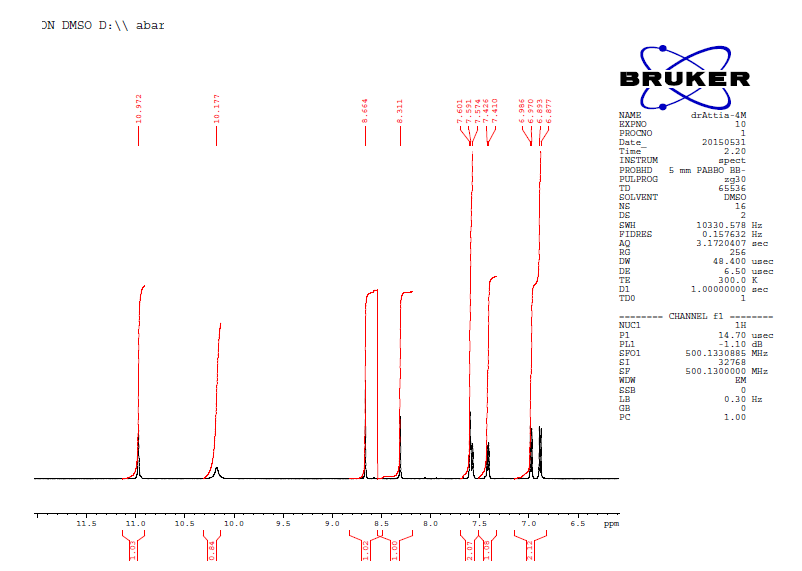


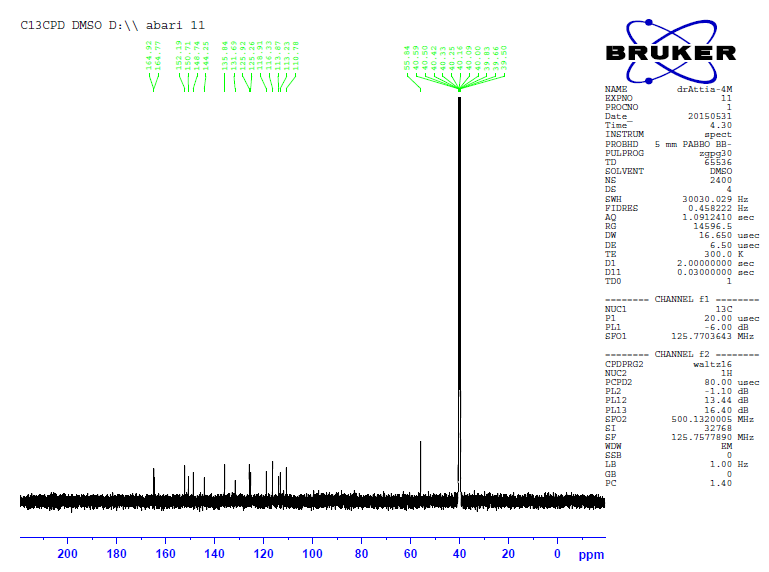


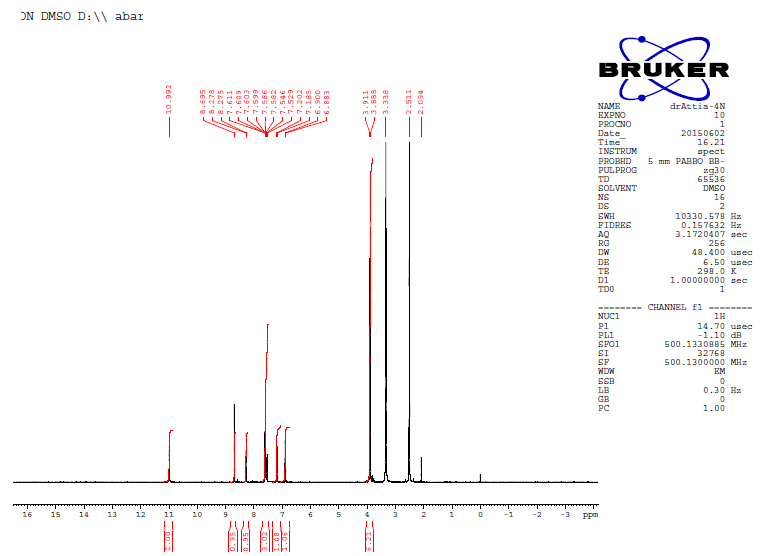


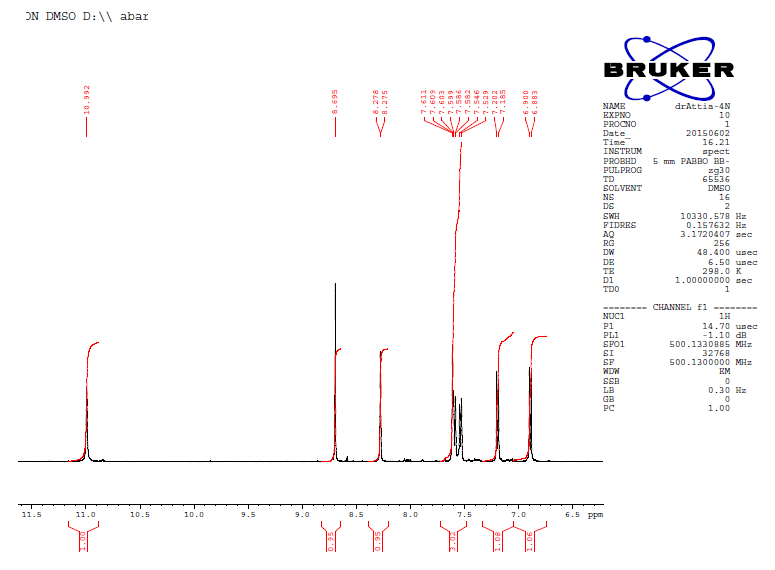


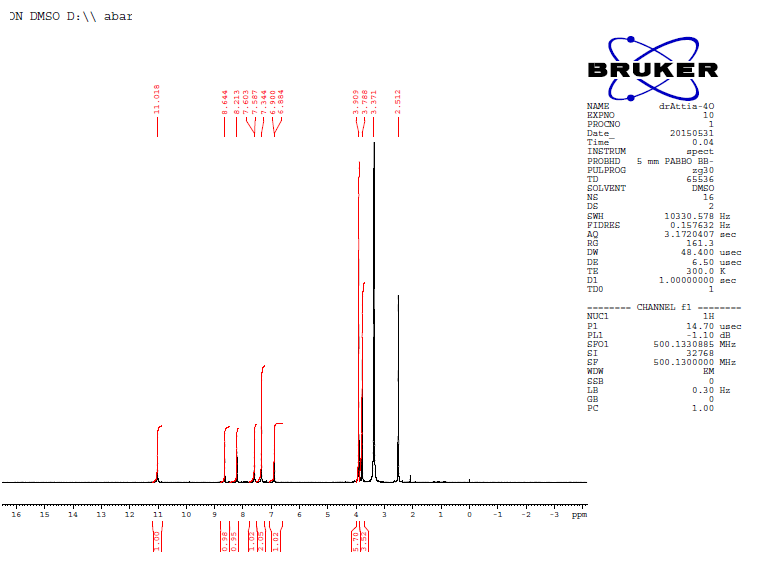


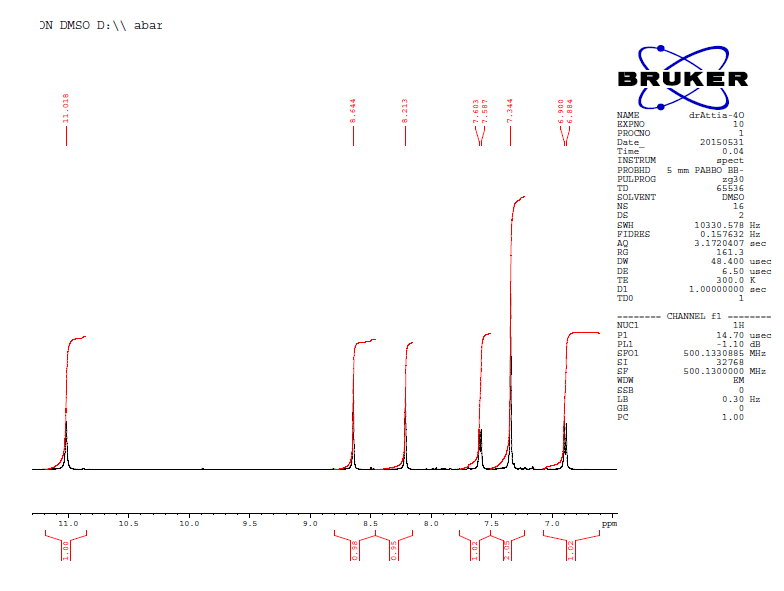


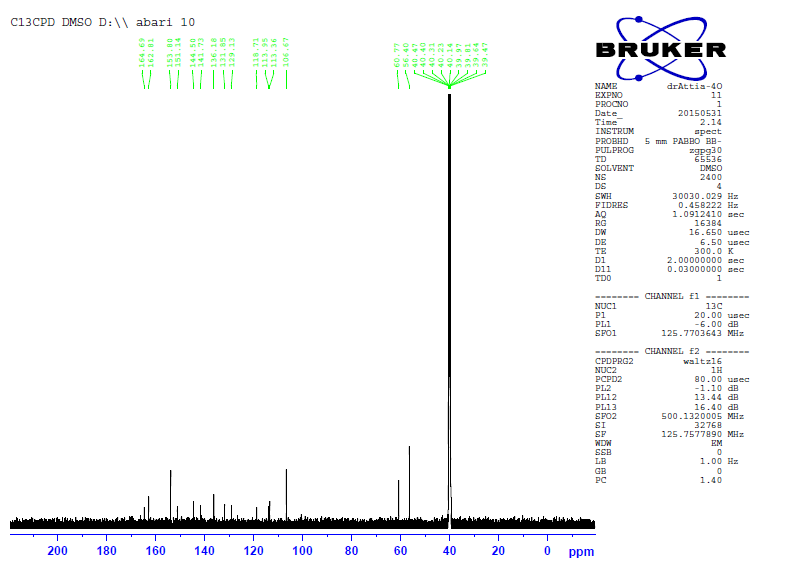


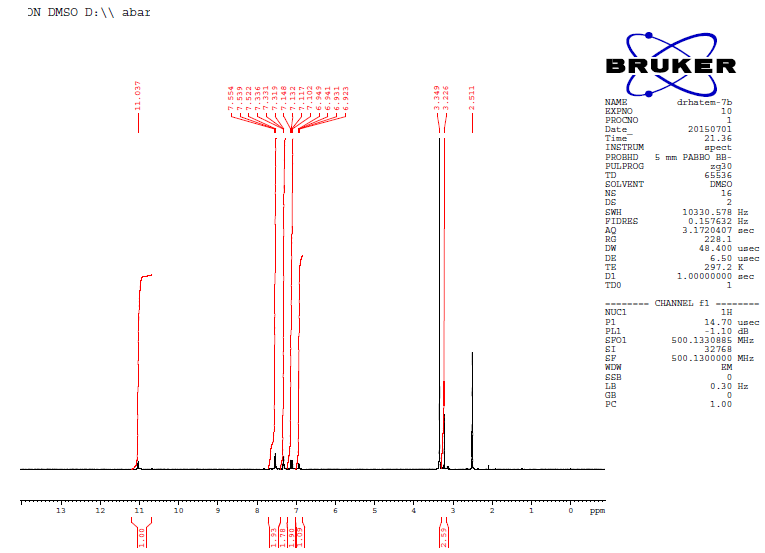


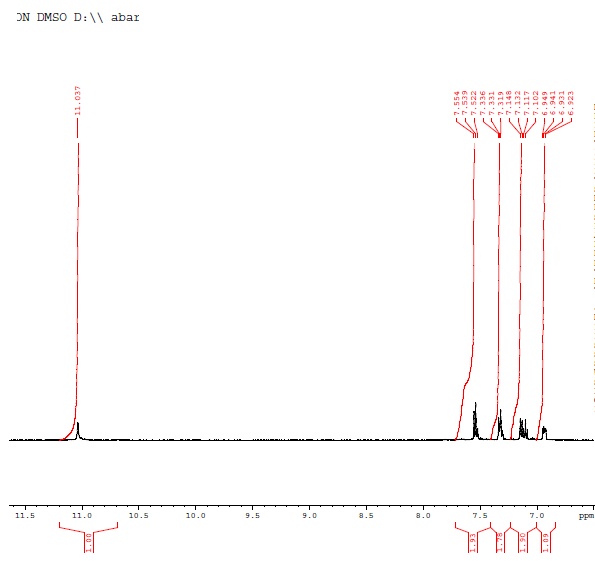


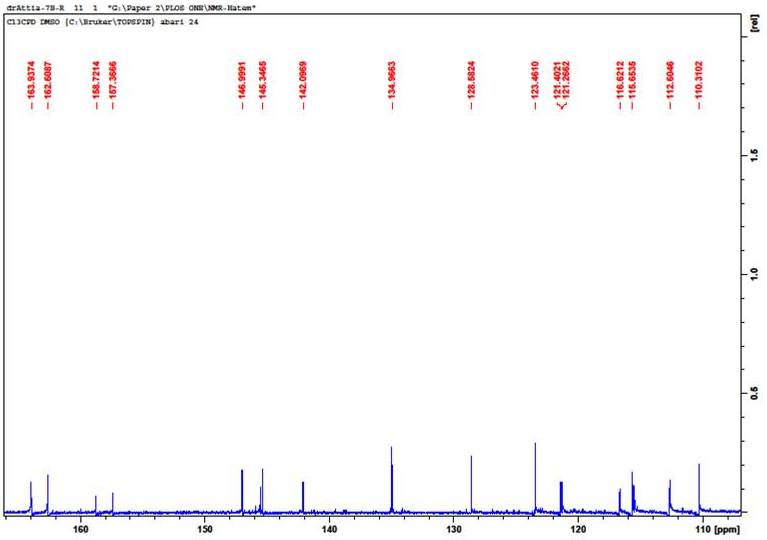


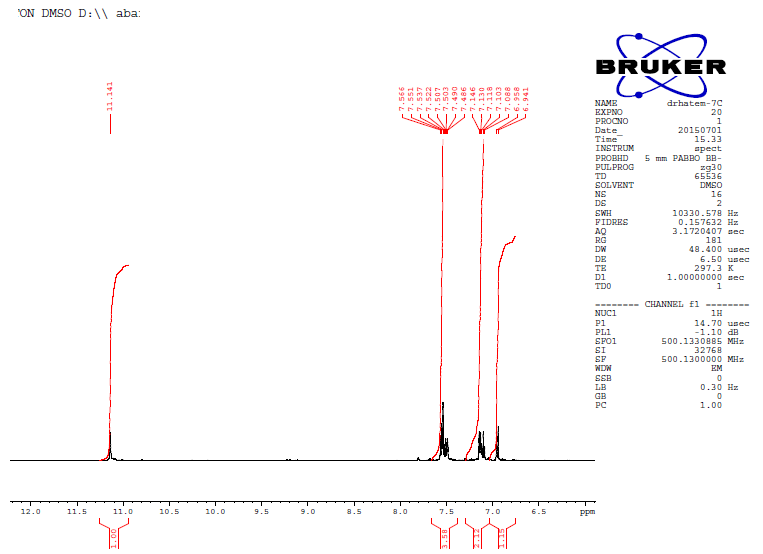


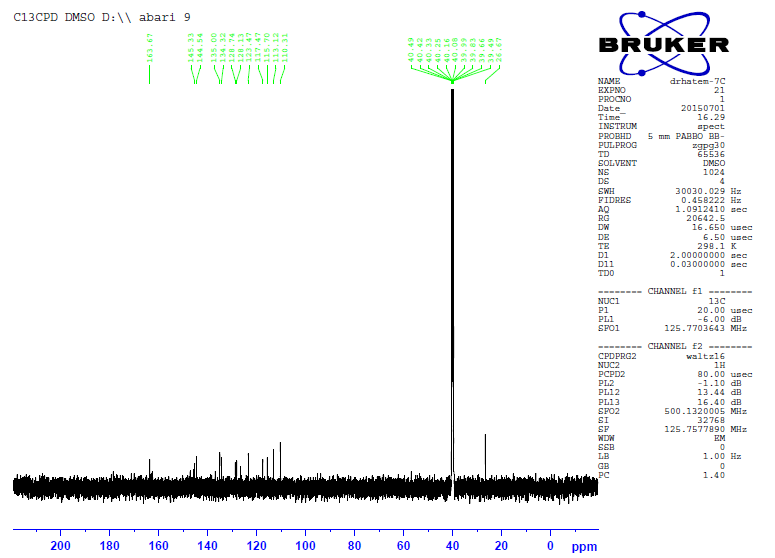


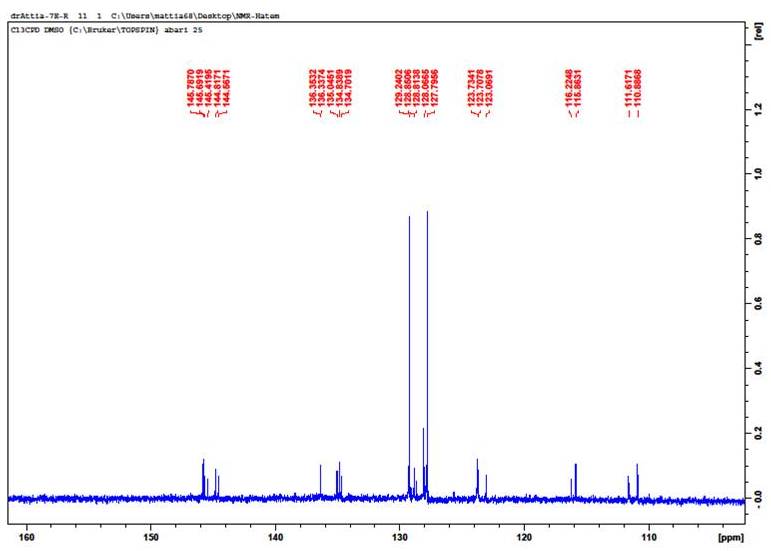


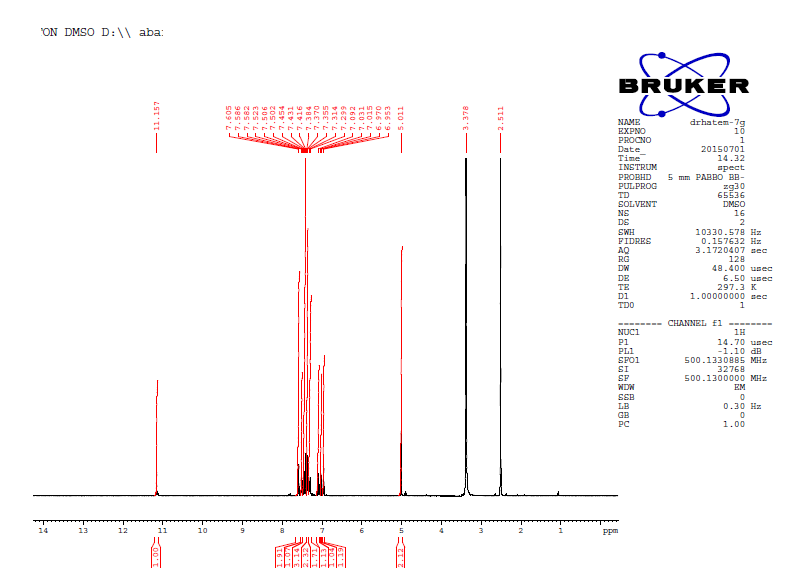


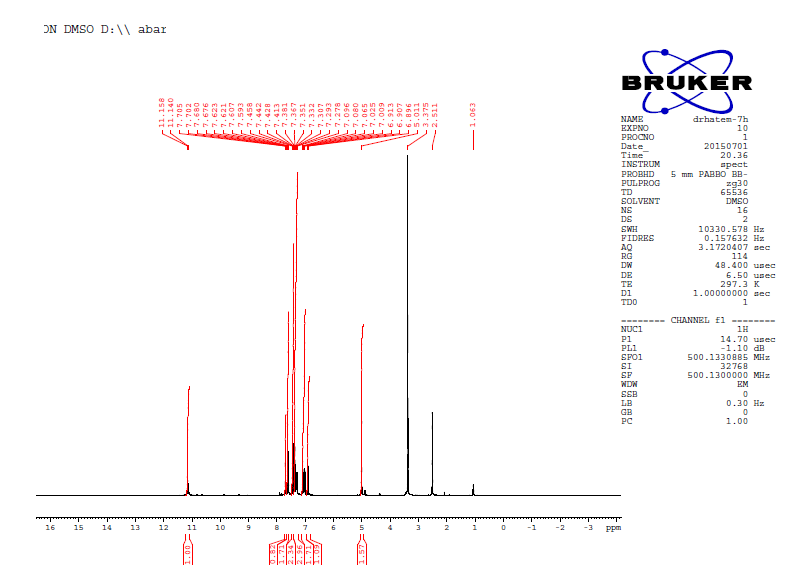


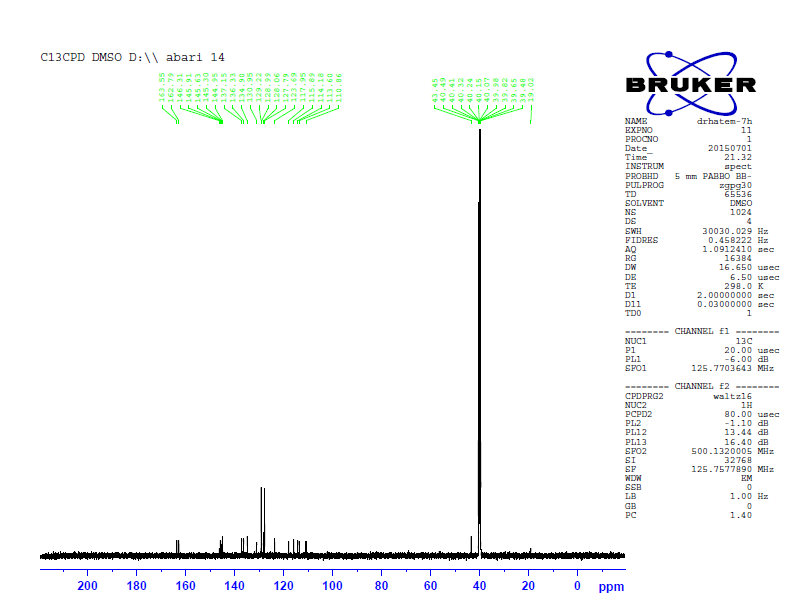

Supplement: S2 File — (DOC) [file pone.0181241.s002.doc]
