# Supplementary material for: New hydrazonoindolin-2-ones: Synthesis, exploration of the possible anti-proliferative mechanism of action and encapsulation into PLGA microspheres
Source: PLoS One. 2017 Jul 25;12(7):e0181241. doi: 10.1371/journal.pone.0181241 (PMC5526551; doi:10.1371/journal.pone.0181241)
Supplement: Supplementary file 3 [file pone.0181241.s003.docx]

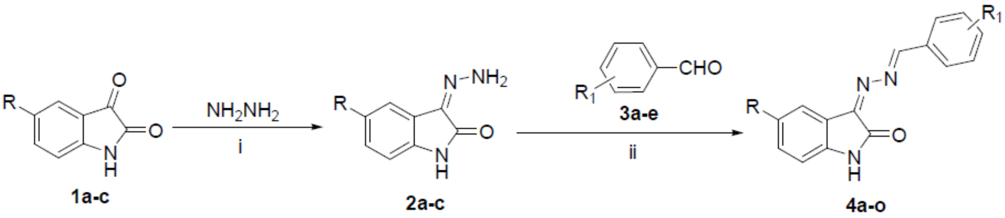

4	R	R_1
a	H	4-OCH_3
b	H	3-OH^{-4}-OCH_3
c	H	4-OH^{-3}-OCH_3
d	H	3,4^{-}$(OCH_3)_2$
e	H	3,4,5^{-}$(OCH_3)_3$
f	Cl	4-OCH_3
g	Cl	3-OH^{-4}-OCH_3
h	Cl	4-OH^{-3}-OCH_3
i	Cl	3,4^{-}$(OCH_3)_2$
j	Cl	3,4,5^{-}$(OCH_3)_3$
k	Br	4-OCH_3
l	Br	3-OH^{-4}-OCH_3
m	Br	4-OH^{-3}-OCH_3
n	Br	3,4^{-}$(OCH_3)_2$
o	Br	3,4,5^{-}$(OCH_3)_3$

Scheme 1. **Synthetic pathway to achieve the target compounds 4a-o.** *Reagents and conditions*: (i) EtOH/ reflux 1 h; (ii) EtOH/AcOH (catalytic) / reflux 4 h.
