# Supplementary material for: New hydrazonoindolin-2-ones: Synthesis, exploration of the possible anti-proliferative mechanism of action and encapsulation into PLGA microspheres
Source: PLoS One. 2017 Jul 25;12(7):e0181241. doi: 10.1371/journal.pone.0181241 (PMC5526551; doi:10.1371/journal.pone.0181241)
Supplement: Supplementary file 4 [file pone.0181241.s004.docx]

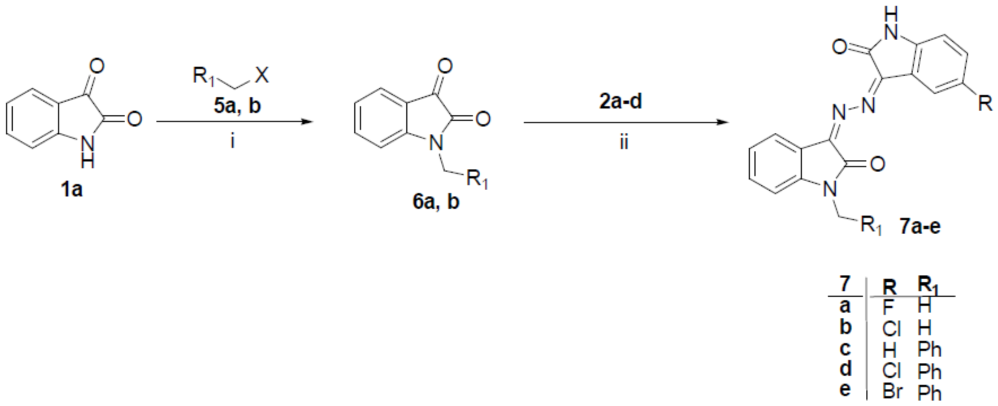


**Scheme 2**. **Synthesis of the target compounds 7a-e.** *Reagents and conditions*: (i) Acetonitrile / $K_2CO_3$ / reflux 4 h; (ii) EtOH/AcOH (catalytic) / reflux 6 h.
